# Transport Coherence Loss in Heterogeneous Forward Osmosis Membranes: A Hierarchical Diagnostic Framework

**DOI:** 10.3390/membranes16060211

**Published:** 2026-06-18

**Authors:** Maurizio Viviani, Nicola Luigi Bragazzi, Gaositwe Bolani, Simonetta Papa, Luca Giacomelli, Roberto Eggenhöffner

**Affiliations:** 1 Robotics.it LLC, 9649 West Olympic Blvd, Suite 7, Beverly Hills, CA 90212, USA; 2 Laboratory for Industrial and Applied Mathematics (LIAM), Department of Mathematics and Statistics, York University, Toronto, ON M3J 1P3, Canada; 3 Clinical Pathology, School of Pathology, Faculty of Health Sciences, University of the Witwatersrand, Johannesburg 2193, South Africa; 4 Polistudium SrL, Via Solferino 7, 20121 Milano, Italy

**Keywords:** forward osmosis, transport coherence, reverse solute flux, pore-size heterogeneity, chemistry–geometry coupling, defect-mediated transport, pore-size distribution, nanostructured membranes

## Abstract

Forward osmosis (FO) membranes are commonly evaluated through macroscopic observables such as water flux and reverse solute flux. However, these quantities do not necessarily reveal whether water permeation and solute leakage remain governed by the same dominant transport pathways, particularly in heterogeneous nanostructured membranes where selective nanochannels and defect-mediated pores can contribute differently to solvent and solute transport. Here, we introduce a hierarchical diagnostic framework to assess transport coherence loss in heterogeneous FO membranes. The framework comprises a baseline model (BM), an extended model (EM) including chemistry–geometry coupling through accessibility loss, and a full model (FM) incorporating selective pore-size heterogeneity. The ratio of reverse solute flux to water flux $R_{J}=J_{s}/J_{w}$ is used as a regime-based diagnostic descriptor of transport organisation, while its normalised form maps coherence variations across the state-space defined by structural selectivity and nanochemical state. The results show that chemistry–geometry coupling produces the first clear reorganisation of the coherence landscape, whereas pore-size heterogeneity mainly broadens the response while preserving its dominant topology. Simulations based on both Monte Carlo and experimentally derived pore-size distributions show consistent trends. Overall, the BM–EM–FM hierarchy offers an interpretable framework for describing transport coherence loss and the emergence of leakage-prone regimes in heterogeneous FO membranes.

## 1. Introduction

Forward osmosis (FO) is a membrane process in which water permeates across a semi-permeable barrier in response to the osmotic gradient between feed and draw solutions [1,2,3]. Because FO operates without the high hydraulic pressures typical of reverse osmosis, it has attracted sustained interest for desalination, concentration, resource recovery, and hybrid treatment systems. FO membrane performance is commonly assessed through macroscopic observables, such as water flux and reverse solute flux, which are typically interpreted through permeability-based or phenomenological transport models [3,4,5,6].

Within nanostructured selective layers, water and solute transport depend on the coupled effects of pore geometry, surface chemistry, confinement, and interfacial interactions [7,8,9,10,11,12]. Here, surface chemistry refers to local physicochemical features of the selective pathways, including charge development, oxidation state, hydration, swelling, and partial obstruction, which can modify both ion exclusion and effective pathway accessibility. The limitation addressed in this work is that macroscopic fluxes alone do not necessarily identify whether water and solute transport remain governed by the same effective pathway population. Although formulated at a general membrane level, the present investigation is primarily motivated by nanostructured FO selective layers, especially graphene- and graphene-oxide-related systems. In nanostructured membranes, marked departures from classical continuum expectations are produced by slip-enhanced flow, steric exclusion, hydration, among others, and electrostatic interactions may contribute simultaneously to transport selectivity [7,9,10,11,13,14,15]. Under sufficiently selective conditions, highly confined channels may sustain substantial water transport while strongly attenuating ion permeation [8,9,11]. However, in heterogeneous membrane networks, similar macroscopic fluxes do not necessarily imply that water permeation and solute leakage continue to sample the same dominant internal transport pathways [9,10,16,17].

Real FO membranes are heterogeneous transport networks rather than perfectly uniform selective nanochannels [10,16,17,18]. Variations in pore size, interlayer spacing, local chemistry, and defect density generate pathway populations with different hydraulic and diffusive properties. In graphene-oxide and other lamellar membranes, highly selective sub-nanometric channels may coexist with wider or weakly selective defects, so that water and solute transport do not necessarily probe the same effective membrane structure [9,16,17]. Even a relatively small population of larger or weakly selective pathways may contribute disproportionately to solute leakage while affecting hydraulic transport much less strongly. Under these conditions, conventional macroscopic indicators considered in isolation may fail to reveal whether transport remains governed predominantly by a selective osmotic network or is progressively redistributed toward leakage-prone routes [9,10,16,17].

A substantial body of work has clarified how nanoscale structure and chemistry affect ion rejection and transport in graphene-based and related nanostructured membranes, with important implications for desalination and selective separations more broadly [8,9,10,13,14,15]. Many FO transport models, however, still represent the membrane through an effective architecture in which water and solute transport are implicitly assumed to sample the same dominant pathway population. Although this approximation may be adequate for structurally uniform selective layers, it becomes increasingly fragile when heterogeneous pathways coexist and contribute unequally to solvent permeation and reverse solute leakage [16,17,18]. In such cases, acceptable macroscopic performance does not necessarily imply that the underlying transport organisation remains predominantly selective [9,10,16,17,18].

To address this problem, the present work introduces a framework for evaluating the coherence of solvent and solute transport in heterogeneous FO membranes. The membrane is represented as a coarse-grained pathway network composed of two parallel classes of conductive routes: selective nanochannels and defect-mediated pores. Within this representation, the measurable responses remain the water flux $J_{w}$ and the reverse solute flux $J_{s}$, while their coupled balance is interpreted through the ratio $R_{J}=J_{s}/J_{w}$. Here, $R_{J}$ is used not as an isolated membrane-performance metric, but as an interpretable diagnostic descriptor of transport coupling and of the extent to which solvent and solute fluxes remain governed by a common effective transport organisation.

To examine how structural heterogeneity and nanochemical modulation reshape this coupling, we develop a hierarchical BM–EM–FM sequence of increasing structural realism. The baseline model (BM) describes the reference redistribution of transport under fixed selective geometry. The extended model (EM) introduces chemistry-dependent renormalisation of selective accessibility, thereby capturing hydration, swelling, partial obstruction, and related chemistry–geometry coupling effects. The full model (FM) extends this formulation to heterogeneous pore populations by incorporating pore-size distributions derived from both Monte Carlo sampling and experimentally reported pore statistics. The resulting responses are analysed as coherence landscapes in the state-space defined by structural selectivity and nanochemical state.

This study aims to determine when water permeation and reverse solute leakage in FO remain governed by the same dominant selective pathway population, and when they progressively decouple as transport redistributes across heterogeneous membrane routes. In this context, transport coherence does not denote ideal selectivity; rather, it denotes the persistence of a common effective transport organisation for solvent and solute transport through the same dominant selective pathway population. This issue is especially relevant in heterogeneous nanostructured FO membranes, where similar water fluxes may mask substantial differences in the internal pathway populations sustaining reverse solute leakage [9,10,16,17]. On this basis, the BM–EM–FM hierarchy is used to separate the effects of chemistry–geometry coupling from those of pore-size heterogeneity and to identify how leakage-prone transport regimes emerge and evolve as the selective pathway population progressively loses accessibility. The framework is intended as an interpretable diagnostic model rather than as a membrane-specific predictive tool.

## 2. Model Framework and Numerical Implementation

FO transport is described here through a coarse-grained diagnostic framework in which the selective layer is represented as a pathway network linking channel-scale geometry and local chemistry to the macroscopic responses given by the water flux $J_{w}$ and the reverse solute flux $J_{s}$. The behaviour of these observables is evaluated using the coherence ratio, which serves as a diagnostic tool for assessing whether the same predominant pathway population regulates water permeation and reverse solute leakage. This formulation retains the essential competition between selective and leakage transport while avoiding atomistic detail and provides the level of description required to examine how geometry, nanochemistry, and pore-size heterogeneity reshape the internal transport state of heterogeneous FO membranes. The model hierarchy introduced below progresses from the BM to the EM and the FM, thereby isolating the effects of chemistry–geometry coupling and pore-size heterogeneity within a common framework [1,2,19].

### 2.1. Transport Framework and Coherence Indicator

The selective layer is represented as a conductive network composed of two parallel pathway populations: selective nanochannels, which support coupled water transport and solute exclusion, and defect-mediated pores, which provide weakly selective leakage routes. Within this representation, the total water and solute fluxes are written as:

(1) $$J_{w}=G\;J_{w,s}+\left(1-G\right)\;J_{w,d}$$

(2) $$J_{s}=G\;J_{s,s}+\left(1-G\right)\;J_{s,d}$$

where $J_{w,s}$ and $J_{s,s}$ denote the selective contributions, $J_{w,d}$ and $J_{s,d}$ are the defect-mediated contributions, and G is an effective structural weighting factor. In the present formulation, G is interpreted as a coarse-grained descriptor of selective-network dominance rather than as a literal geometrical area fraction.

As introduced above, we define the coherence ratio to track the coupled balance between solvent permeation and reverse solute leakage:

(3) $$R_{J}=\frac{J_{s}}{J_{w}}$$

Low and smoothly varying values of $R_{J}$ identify transport states in which solvent permeation and reverse solute leakage remain predominantly coupled through the selective network, whereas increasing values indicate progressive redistribution of solute transport toward weakly selective or defect-mediated routes.

For comparisons across nanochemical states, a normalised form is introduced:

(4) $$\tilde{R_{J}}\left(G,\chi \right)=\frac{R_{J}\left(G,\chi \right)}{R_{J}\left(G,0\right)}$$

where χ is the nanochemical activation parameter describing the progressive modification of the selective population by oxidation, charge development, hydration, local swelling, or partial obstruction [16,20]. In this normalised representation, $\tilde{R_{J}}$ isolates the relative effect of chemistry at fixed structural selectivity and provides a direct map of coherence variation within the state-space.

### 2.2. Pathway-Scale Transport Relations

Pathway-scale transport is represented through reduced constitutive relations for water flow and solute diffusion that retain the dominant dependencies on pathway radius, transport length, interfacial slip, steric confinement, and electrostatic exclusion,

(5) $$J_{w}^{\left(path\right)}=n_{p}\frac{\pi r^{4}}{8\mu L}\left(1+\frac{4b}{r}\right)\Delta p_{eff}$$

where $n_{p}$ is the number of active pores per unit membrane area, L is the characteristic transport length, μ is the dynamic viscosity of water $(1.0\times 10^{-3}\;Pa\cdot s),\;b$ is the slip length at the channel wall, and $\Delta p_{eff}$ is a pressure-equivalent representation of the effective chemical-potential driving force for water permeation. The use of the bulk dynamic viscosity is an effective continuum-level approximation adopted to preserve a tractable pathway-scale description. Possible nanoscale deviations from classical viscous behaviour are not resolved explicitly; instead, their leading influence is incorporated phenomenologically through the slip-length term. Equation (5) defines the pathway-scale hydraulic relation used throughout the BM–EM–FM hierarchy, retaining the leading dependence of conductance on pore size, transport length, and interfacial slip. In this framework, selective nanochannels are associated with larger slip enhancement, whereas defect-mediated pathways are assigned smaller slip lengths, consistent with previous experimental and theoretical observations [8,21,22]. The solute flux along a pathway is expressed as

(6) $$J_{s}^{\left(path\right)}=n_{p}\;\pi \;r^{2}\frac{D_{0}}{L}\;K_{steric}\left(\lambda \right)\;K_{elec}\left(\chi \right)\;\Delta c$$

where $D_{0}$ is the bulk diffusion coefficient, Δc is the concentration difference across the membrane, and $K_{steric}$ and $K_{elec}$ are the steric and electrostatic attenuation factors defined below [7,9]. Equation (6) provides the pathway-scale molar solute flux per unit membrane area; the factor $n_{p}\pi r^{2}$ represents the total active diffusive cross-section associated with the pore population, so that Equation (6) preserves the leading dependence of solute transport on pathway radius, transport length, steric accessibility, and chemistry-dependent exclusion within the BM–EM–FM framework. Here, r denotes the effective pathway radius used in the corresponding transport branch. In the selective branch, $r=r_{s}$ in BM and $r=r_{s,eff}$ in EM/FM; in the defect-mediated branch, $r=r_{d}$. The corresponding reference values and units are reported in Table 1.

Steric exclusion is expressed in terms of the solute-to-pore size ratio,

(7) $$\lambda =\frac{a}{r}$$

where a is the effective hydrated solute radius. Here, r denotes the effective pathway radius, whereas a denotes the effective hydrated solute radius entering the steric exclusion term; the two quantities therefore refer to different physical objects. The steric term is written as

(8) $$K_{steric}(\lambda )=(1-\lambda {)}^{2}$$

so that solute entry is progressively suppressed as the accessible pore radius approaches the hydrated solute size [7]. Equation (8) holds for 0≤λ≤1, while for λ≥1, $K_{steric}$ vanishes. For selective channels, electrostatic attenuation is represented by

(9) $$K_{elec}\left(\chi \right)=e^{-\alpha \chi }$$

where α controls the strength of chemistry-dependent exclusion and χ is the nanochemical activation parameter [20,22,23]. In defect-mediated pathways, steric and electrostatic attenuation are assumed to be weak at leading order, so that solute transport approaches near-bulk diffusive behaviour. This asymmetry allows the selective and defect-mediated branches to respond differently to the same nanochemical perturbation while remaining embedded in a common pathway-scale transport framework.

### 2.3. Hierarchy of Model Formulations

Starting from the common transport framework above, three model levels are introduced to isolate baseline redistribution, chemistry-dependent accessibility loss, and pore-size heterogeneity.

In the BM, channel geometry is kept fixed, and chemistry acts only through interfacial slip and electrostatic attenuation [7,21,22]. The selective radius is therefore treated as constant,

(10) $$r_{s,eff}=r_{s}$$

so that BM provides the reference coherence field before explicit chemistry–geometry coupling is introduced.

In the EM, chemistry is also allowed to modify selective accessibility [16,20,22,23]. This is introduced through a chemistry-dependent contraction of the selective radius,

(11) $$r_{s,eff}=r_{s}\left(1-\beta \chi \right)$$

where β controls the strength of chemistry–geometry coupling. To preserve physical admissibility, $r_{s,eff}$ is constrained to remain positive over the explored parameter range. In parallel, the functional weight of the selective branch is attenuated as

(12) $$G_{eff}=G\left(1-\gamma \chi \right)$$

with clipping to [0,1], where γ quantifies the progressive loss of functionally accessible selective pathways. In the reduced BM–EM description, the selective branch is therefore represented by a single effective selective radius.

Within EM, Equation (11) acts on the effective selective radius and therefore modifies geometric accessibility at the pathway level, whereas Equation (12) attenuates the functional weight of the selective branch at the network level. The two terms thus represent distinct but coupled effects of nanochemical activation.

In the FM, the deterministic EM formulation is extended to a heterogeneous selective population. FM preserves the EM transport architecture but replaces the single reduced selective radius with a distributed selective pore population p(r), so that the selective contribution is evaluated as a distribution-averaged response under the same nanochemical state. The corresponding distribution-based extension is introduced in Section 2.4.

### 2.4. Pore-Size Distributions and FM Implementation

The FM replaces the single effective selective radius with a normalised selective pore-size distribution $p\left(r\right)$, thereby accounting for structural variability within the selective pathway network [3,10,11,17,18,24,25]. In this way, FM preserves the EM architecture while allowing different members of the selective population to contribute differently to water and solute transport under the same nanochemical state.

In EM, the total fluxes are written as

(13) $$J_{w}^{EM}=G_{eff}\;J_{w,s}\left(r_{s,eff},\chi \right)+\left(1-G_{eff}\right)\;J_{w,d}$$

(14) $$J_{s}^{EM}=G_{eff}\;J_{s,s}\left(r_{s,eff},\chi \right)+\left(1-G_{eff}\right)\;J_{s,d}$$

where the selective contribution is evaluated at the chemistry-modified effective radius, while the defect-mediated branch remains represented by an effective defect pathway.

In FM, the selective contribution is evaluated as an ensemble average over the distributed radius population, with

(15) $$\int \;p\left(r\right)\;dr=1$$

and the macroscopic fluxes become

(16) $$J_{w}^{FM}=G_{eff}\int \;J_{w,s}\left(r,\chi \right)\;p\left(r\right)\;dr+\left(1-G_{eff}\right)\;J_{w,d}$$

(17) $$J_{s}^{FM}=G_{eff}\int \;J_{s,s}\left(r,\chi \right)\;p\left(r\right)\;dr+\left(1-G_{eff}\right)\;J_{s,d}$$

These expressions preserve EM closure while replacing the deterministic selective response with a distribution-averaged one, thereby allowing the effect of selective pore-size heterogeneity to be evaluated within the same transport framework.

In the numerical implementation, the integrals are approximated by discrete quadrature around the EM radius,

(18) $$r_{k}=r_{s,eff}\;u_{k}$$

where $u_{k}$ are dimensionless support factors and $w_{k}$ are the associated normalised weights. The resulting discrete formulation is

(19) $$J_{w}^{FM}\approx G_{eff}\sum_{k}w_{k}\;J_{w,s}\left(r_{k},\chi \right)+\left(1-G_{eff}\right)\;J_{w,d}$$

(20) $$J_{s}^{FM}\approx G_{eff}\sum_{k}w_{k}\;J_{s,s}\left(r_{k},\chi \right)+\left(1-G_{eff}\right)\;J_{s,d}$$

This construction ensures continuity between model levels, since the heterogeneous formulation reduces to EM in the narrow-distribution limit,

(21) $$\underset{\sigma_{r}\to 0}{lim}p\left(r\right)=\delta \left(r-r_{s,eff}\right)$$

where *σ_r_* denotes the width of the selective pore-size distribution. This limiting property guarantees that FM acts as a heterogeneity extension of EM rather than as an unrelated third formulation. Two heterogeneous implementations are considered. In the first, $p\left(r\right)$ is generated synthetically through Monte Carlo sampling in order to isolate the generic effect of structural heterogeneity. In the second, $p\left(r\right)$ is derived from experimentally reported pore-size distributions for nanostructured FO membranes [3,10,11,17,18,24,25]. In that case, literature histograms or probability density functions are imported, normalised, and interpolated onto the quadrature grid.

### 2.5. Numerical Implementation and Parameter Space

All formulations are evaluated over the state-space defined by the structural selectivity parameter G and the nanochemical state χ, with 0≤G≤1 and 0≤χ≤1. Within this space, water and solute fluxes are computed from the same transport framework and subsequently combined to obtain the coherence indicator $R_{J}$ and, where required, its normalised form $\tilde{R_{J}}$. The primary interpretation domain of the present model is therefore the state-space (G,χ), from which the observable responses $J_{w}$, $J_{s}$, and $R_{J}$ are computed.

Model parameters are selected within physically plausible ranges representative of nanostructured FO membranes [1,2,19,20,22,23]. Although the framework is formulated in general terms for heterogeneous FO membranes, its present parameterisation is most directly motivated by nanostructured systems such as graphene- and graphene-oxide-based selective layers [16,20,23]. The explored ranges include sub-nanometric selective radii, defect radii of a few nanometers, transport lengths of tens of nanometers, large slip lengths for graphene-like interfaces, smaller slip lengths for oxidised or defect-dominated states, and confinement-reduced diffusive transport [8,9,21,22]. A summary of the model parameters, units, and reference values adopted in the present work is reported in Table 1. The reported values define the working parameter set used to generate the transport and coherence landscapes discussed in the present study.

**Table 1:** Representative working parameter set adopted for the BM–EM–FM calculations. The reported values define the executable reference set used to generate the transport and coherence landscapes discussed in the present study. The final column distinguishes between literature-based reference inputs, literature-informed working values, framework coefficients, and internal state-space variables. For parameters not uniquely fixed by external measurements, the associated robustness role is stated and discussed in Supplementary Materials Section 2.

Category	Symbol	Description	Unit	Reference Value	Provenance/Robustness Role
Geometry	$r_{s}^{BM/EM}$	Effective selective transport radius	nm	**0.5**	Fang 2014 [26]; Kim 2017 [27]
Geometry	$p\left(r\right)/r_{s}^{FM}$	Selective pore-size distribution	nm	See Section 3.3.1	Fang 2014 [26]; Kim 2017 [27]
Geometry	***r_d_***	Effective defect-path radius	nm	1.500	O’Hern et al. 2012 [28]
Geometry	***L_s_***	Effective selective-path length	nm	100	Literature-informed working value; secondary sensitivity tested in Supplementary Materials Section 2
Geometry	***L_d_***	Effective defect-path length	nm	100	Literature-informed working value; secondary sensitivity tested in Supplementary Materials Section 2
Hydro-dynamics	***b_s_***	Slip length in selective channels	nm	20	Literature-informed working value; secondary sensitivity tested in Supplementary Materials Section 2
Hydro- dynamics	***b_d_***	Slip length in defect pathways	nm	10	Literature-informed working value; secondary sensitivity tested in Supplementary Materials Section 2
Driving term	***Δp_eff_***	Effective pressure-equivalent driving term	Pa	1.00 × 10^6^	Executable reference scale; robustness discussed in Supplementary Materials Section 2
Diffusion	***Δc***	Reference concentration driving term	mol·m^−3^	1000	Executable reference scale; robustness discussed in Supplementary Materials Section 2
Diffusion	***D*** **_0_**	Representative ionic reference bulk diffusivity in water	m^2^·s^−1^	1.6 × 10^−9^	Lobo 1989 [29]
Sterics	***a***	Effective hydrated solute radius	nm	0.325	Abraham 2017 [30]; Joshi 2014 [10,11]; Lancellotti 2024 [13]
Coupling	***α***	Electrostatic attenuation coefficient linked to the literature electrostatic exclusion scale	–	2.2	Framework coefficient; electrostatic robustness tested in Supplementary Materials Section 2
Coupling	***β***	Dimensionless chemistry–geometry coupling coefficient controlling chemistry-induced contraction of the selective-path radius	–	0.20	Framework coefficient; chemistry–geometry coupling tested in Supplementary Materials Section 2
Coupling	***γ***	Dimensionless selective-accessibility attenuation coefficient controlling chemistry-induced loss of effective selective contribution	–	0.35	Framework coefficient; selective-accessibility robustness tested in Supplementary Materials Section 2
State-space	***G***	Structural selectivity state variable spanning the relative dominance of selective over defect-mediated transport	0–1	0–1	Internal state variable; structural selectivity coordinate
State-space	***χ***	Nanochemical state variable controlling chemistry-dependent attenuation and accessibility loss	0–1	1	Internal state variable; nanochemical state coordinate

The parameters reported in Table 1 define the representative working set adopted for the BM–EM–FM calculations. They are treated as bounded mechanistic inputs selected within physically plausible ranges for nanostructured FO membranes and are intended to represent a generic nanoscale transport system rather than a specific membrane architecture. Selective pathways correspond to sub-nanometric transport channels responsible for coherent osmotic water transport, whereas defect-mediated pathways represent larger, weakly selective leakage routes that primarily contribute to reverse solute transport. Surface chemistry modifies the selective branch through the effective-radius relation in Equation (11) and through the attenuation terms introduced in Section 2.3, while defect-path geometry is treated, at leading order, as comparatively insensitive to chemistry. Hydrodynamic slip is represented through effective slip lengths assigned to selective and defect pathways, and steric confinement is controlled by the hydrated solute radius entering the hindrance relation.

For the Monte Carlo FM implementation, selective radii are sampled from a truncated log-normal distribution centered on the EM radius, and each state is evaluated over repeated realisations. In the present implementation, heterogeneity is introduced through the selective pore-radius population only. Ensemble statistics include the mean, standard deviation, and coefficient of variation of $R_{J}$ in order to quantify the amplification of transport variability induced by structural heterogeneity. For the experimentally derived FM implementation, literature pore-size distributions are imported as normalised histograms or probability density functions and used directly as p(r) after interpolation onto the numerical quadrature grid. Unless otherwise stated, results are reported in normalised form to compare chemistry- and heterogeneity-induced redistribution across the state-space on a common basis.

### 2.6. Scope and Limitations of the Present Framework

The present framework provides a membrane-level diagnostic description of how forward osmosis transport reorganizes as selective accessibility is progressively lost in a heterogeneous membrane network.

Throughout the diagnostic description, the purpose of the present framework is to identify the conditions under which water permeation and reverse solute leakage remain governed predominantly by the same effective selective pathway population, and the conditions under which they progressively decouple as leakage-prone routes become functionally more relevant. The parameters are selected within physically plausible ranges representative of nanostructured FO membranes, but they are not calibrated to reproduce the performance of a single material under a unique set of operating conditions. The resulting transport landscapes should therefore be interpreted as responses that resolve pathway competition, transport reorganisation, and heterogeneity effects, rather than as point-by-point predictions of absolute membrane performance.

The formulation isolates the pathway-scale mechanisms of interest by maintaining a tractable membrane-level description of selective-versus-defect-mediated transport competition. Other device-level processes, such as support-layer resistance, fouling, compaction, and ageing are not included here because they require membrane-specific time-resolved descriptions outside the present diagnostic scope. As an exploratory extension, the FM generator was additionally evaluated after incorporating external and internal concentration polarisation corrections, following the formulations of McCutcheon and Elimelech [31] and Tan and Ng [32].

The membrane itself is represented through two coarse-grained pathway populations, namely a selective population and a defect-mediated population. This abstraction is sufficient to capture the dominant competition between selective and leakage-prone transport, although real membranes may exhibit a broader continuum of partially selective environments and spatially correlated defects that are not explicitly resolved here. Experimental comparisons are therefore interpreted as tests of physical plausibility and transport ordering rather than as full membrane-specific validation.

## 3. Results

The BM–EM–FM hierarchical framework is used to describe how heterogeneous forward osmosis membranes respond to transport processes. The analysis is organised according to increasing model complexity. We first compare the BM and the EM to isolate the effect of chemistry–geometry coupling on the transport response and on the associated coherence structure. We then examine how pore-size heterogeneity modifies this behaviour within the FM, first through Monte Carlo-generated pore populations and subsequently through an experimentally derived pore-size distribution. Rather than considering only absolute flux magnitudes, the analysis focuses on the coupled behaviour of water flux $J_{w}$, solute flux $J_{s}$, and their ratio $R_{J}$. The results are interpreted primarily through transport and coherence landscapes in the state-space defined by the structural selectivity parameter G and the nanochemical state χ.

### 3.1. BM–EM Comparison: Chemistry–Geometry Coupling and Coherence Reorganisation

#### 3.1.1. Transport-Landscape Reorganisation in the BM and EM

The comparison between the BM and the EM isolates how chemistry–geometry coupling reshapes the transport response across the (G,χ) state-space. In both formulations, the total fluxes arise from the combined contribution of selective nanochannels and defect-mediated pathways, but the two models differ in how nanochemical activation interacts with selective accessibility. In BM, the selective radius is kept fixed, so that chemistry affects transport primarily through interfacial parameters such as slip and electrostatic attenuation. In EM, by contrast, increasing nanochemical activation also reduces the effective accessibility of the selective population. This introduces explicit chemistry–geometry coupling and modifies not only the magnitude but also the topology of the transport fields.

**Figure 1:**
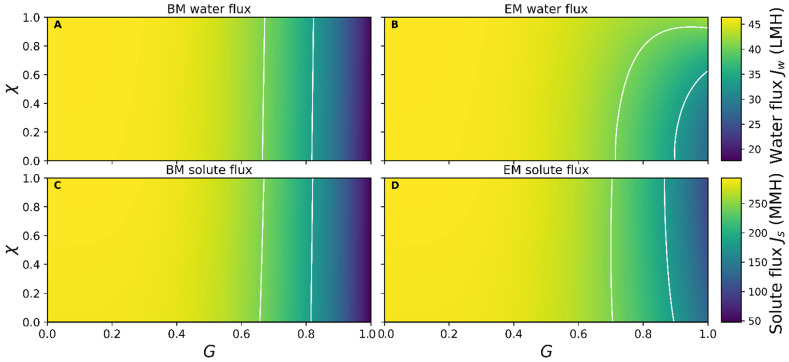
Scaled water- and solute-transport landscapes described by the baseline model (BM) and the extended model (EM). The plotted fields are shown as functions of the structural selectivity parameter G and the nanochemical state χ. White contour lines indicate selected iso-response levels within each transport landscape. (**A**): water flux $J_{w}$ from BM, (**B**): water flux $J_{w}$ from EM, (**C**): solute flux $J_{s}$ from BM, (**D**): solute flux $J_{s}$ from EM. The color scale reports the units of L m^−2^ h^−1^ (LMH) and mmol m^2^ h^−1^ (MMH) for $J_{w}$ and $J_{s},$ respectively.

Figure 1 shows that BM and EM retain the same global monotonic trend while differing markedly in field structure. In BM, both the water-flux and solute-flux maps remain largely structure-dominated: the contour lines are nearly vertical, and the dominant gradient runs primarily along the G direction, indicating that χ acts only as a comparatively weak modulation when selective geometry is fixed. The BM response, therefore, defines the structurally dominated reference field.

In EM, the introduction of chemistry-dependent accessibility loss produces a qualitatively different response, especially in the water-flux field. The EM water-flux map exhibits a pronounced bending of the iso-response contours at high G, showing that once transport becomes dominated by the selective population, increasing χ no longer acts as a minor perturbation but actively reorganises the landscape. Chemistry–geometry coupling, therefore, becomes a genuine state-organising variable rather than a simple amplitude correction.

The solute-flux field is also modified in EM, but the deformation remains appreciably weaker than in the water-flux map under the present parameterisation. Relative to BM, the EM solute-flux landscape remains predominantly G-controlled, with only a moderate curvature of the contour structure. This asymmetry indicates that, in the present model setting, chemistry-dependent accessibility loss perturbs hydraulic transport more strongly than diffusive leakage. A consistent interpretation is that contraction of the selectively accessible population depresses the high-conductance water pathway more visibly, whereas the solute response retains a stronger contribution from the broader leakage background and therefore remains closer to the BM topology.

#### 3.1.2. Coherence-Landscape Reorganisation in the BM and EM

The effect of chemistry–geometry coupling becomes more directly visible when the BM and EM responses are expressed through the normalised coherence ratio $\tilde{R_{J}}$. In this representation, the analysis tracks how the balance between water permeation and solute leakage evolves across the (G,χ) state-space rather than considering the transport fields separately.

**Figure 2:**
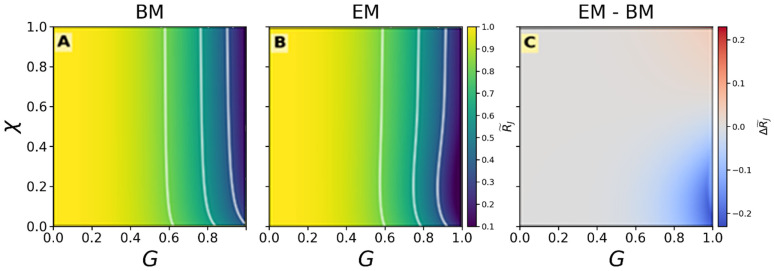
Coherence landscapes in the baseline and extended models. Maps of the normalised coherence ratio $\tilde{R_{J}}\left(G,\chi \right)$ described by the baseline model (BM) (**A**) and the extended model (EM) (**B**) are shown across the $\left(G,\chi \right)$ domain. The right panel reports the corresponding difference field, EM–BM (**C**).

Figure 2 shows that BM defines a smooth reference coherence landscape, with a predominantly regular dependence on both structural selectivity and nanochemical state. Under BM conditions, the normalised field remains comparatively simple because the selective geometry is fixed and the nanochemical contribution acts only through the interfacial transport factors included in the baseline formulation. The resulting map, therefore, reflects the reference redistribution of transport coherence in the absence of explicit chemistry-dependent accessibility loss.

The EM coherence landscape preserves the same global organisation but introduces a more structured response in the high region, where transport becomes increasingly controlled by the selective population. In this regime, the introduction of chemistry-dependent accessibility loss modifies the local balance between water flux and solute leakage more strongly than in weakly selective states. As a result, the coherence field no longer evolves as a smooth perturbation of BM but develops a more localised and state-dependent deformation.

This difference is resolved more clearly in the BM–EM coherence-difference map. The largest deviations are concentrated at high G, indicating that the impact of selective-accessibility loss becomes most visible when the selective branch dominates the overall membrane response. At lower G, where transport already contains a stronger contribution from defect-mediated pathways, the BM and EM landscapes remain comparatively close. The resulting pattern shows that EM does not shift the BM coherence field uniformly, but redistributes transport coherence in a state-dependent manner.

Figure 2, therefore, identifies BM as the reference coherence landscape and EM as a localised reorganisation induced by chemistry–geometry coupling. The transition is best interpreted not as a uniform rescaling of the underlying flux fields, but as a structured perturbation of the coupling between water permeation and solute leakage.

#### 3.1.3. Sectional BM–EM Differences in Normalised Coherence at Fixed Structural Selectivity

Sectional profiles of the BM–EM coherence difference provide a one-dimensional view of how chemistry–geometry coupling modifies normalised transport coherence at fixed levels of structural selectivity. By plotting $\Delta \tilde{R_{J}}(\chi )$ for selected values of G, the analysis resolves directly how the EM perturbation evolves across the nanochemical coordinate within distinct structural-selectivity regimes.

**Figure 3:**
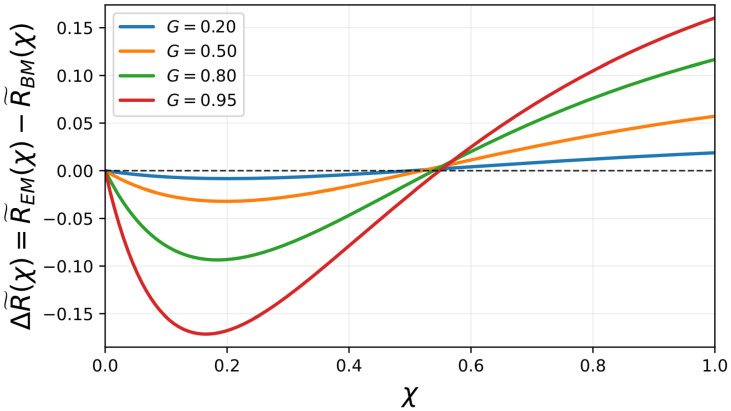
Sectional BM–EM difference in normalised coherence at fixed structural selectivity. The plotted profiles report $\Delta \tilde{R_{J}}\left(\chi \right)={\tilde{R_{J}}}_{EM}\left(\chi \right)-{\tilde{R_{J}}}_{BM}\left(\chi \right)$ at selected values of the structural selectivity parameter G. This representation provides the one-dimensional counterpart of the EM–BM difference field shown in Figure 2 and highlights how the effect of chemistry–geometry coupling varies with nanochemical activation within distinct structural-selectivity regimes.

Figure 3 shows that the BM–EM deviation is strongly state-dependent and increases markedly with structural selectivity. At low G, the sectional profiles remain close to zero over most of the χ range, indicating that when the selective population contributes weakly to the overall response, explicit chemistry-dependent accessibility loss has only a limited influence on normalised coherence. As G increases, however, the deviation becomes progressively larger, showing that the EM correction becomes increasingly relevant once transport is dominated by the selective branch.

The sectional trends also reveal that the BM–EM difference is not monotonic along χ. For sufficiently high G, $\Delta \tilde{R_{J}}(\chi )$ is initially negative, indicating that EM supports lower normalised coherence than BM in the weak-to-intermediate nanochemical regime. With further increase in χ, the deviation rises, eventually crossing zero and becoming positive. The EM perturbation, therefore, does not act as a simple upward or downward shift of the BM coherence field, but as a structured redistribution whose sign and magnitude both depend on the local transport state.

The near-common zero crossing of the sectional $\Delta \tilde{R_{J}}(\chi )$ curves is interpreted here as a property of the present normalised formulation and parameter set rather than as a universal feature of FO transport. What remains robust is the overall trend: the BM–EM difference is negligible in weakly selective states, becomes pronounced in strongly selective states, and changes sign along the χ coordinate. Figure 3, therefore, refines the 2D interpretation of Figure 2 by showing explicitly that the EM perturbation is not a uniform rescaling of the BM coherence field, but a state-dependent reorganisation of normalised transport coherence.

### 3.2. Effect of Pore Size Heterogeneity

#### 3.2.1. Mean-Field Effect of Pore-Size Heterogeneity on the Coherence Landscape

The analysis above represents the selective pathway population through a single effective pore radius. The FM removes this simplification by replacing the deterministic selective branch with a pore-size distribution, so that the macroscopic response is obtained by averaging over a heterogeneous selective population. Water and solute transport no longer arise from a single representative selective channel, but from an ensemble of channels with different radii and therefore different hydraulic and diffusive contributions. To isolate the generic effect of structural heterogeneity, FM is first evaluated using Monte Carlo-generated pore populations.

**Figure 4:**
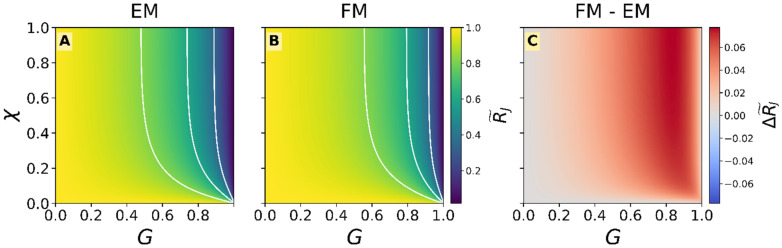
Effect of MC pore-size heterogeneity on the coherence landscape. Comparison between the deterministic extended model (EM) (**A**) and the heterogeneous full model (FM) (**B**) using Monte Carlo-generated pore populations. The left panel shows the normalised coherence field supported by EM, the central panel shows the ensemble-averaged FM response, and the right panel reports the difference field FM–EM (**C**).

Figure 4 shows that pore-size heterogeneity preserves the main topology of the coherence landscape. The EM and FM fields retain the same broad organisation across the $\left(G,\chi \right)$ domain, indicating that the introduction of a distributed selective population does not generate a qualitatively new coherence structure. In this sense, the deterministic EM formulation already captures the dominant state-space organisation, while FM refines it by resolving structural dispersion within the selective branch. In the present Monte Carlo implementation, heterogeneity is introduced through the selective pore-radius population only.

The effect of heterogeneity appears instead through local deviations, highlighted by the FM−EM difference field. These deviations remain limited at low G, where defect-mediated pathways already contribute substantially to the overall transport response, and become progressively more visible as G increases and the membrane state becomes more strongly controlled by the selective population. The influence of Monte Carlo heterogeneity is therefore not uniform across the domain, but is amplified in the high-G regime.

Under the present parameterisation, the FM−EM field is predominantly positive over most of the state-space explored. FM therefore does not simply broaden the EM response symmetrically around the deterministic field; it also introduces a modest upward displacement of normalised coherence, most evident toward high G. This indicates that selective-population averaging preserves the EM topology while slightly shifting the mean coherence response.

Figure 4, therefore, shows that Monte Carlo pore-size heterogeneity does not fundamentally reorganise the coherence landscape. Its principal effect is to preserve the EM topology while introducing structured local deviations whose amplitude becomes more visible as the transport state approaches the strongly selective regime.

#### 3.2.2. Dispersion of the Heterogeneous Response Around the Deterministic Trend

**Figure 5:**
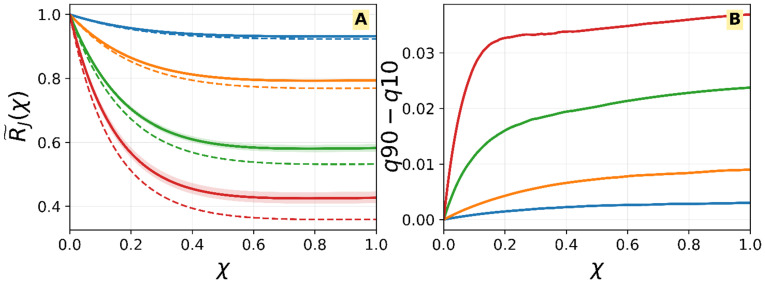
Dispersion of the heterogeneous response around the deterministic trend at fixed structural selectivity. (**A**) compares the deterministic EM sectional profiles of the normalised coherence ratio $\tilde{R_{J}}\left(\chi \right)$ with the corresponding heterogeneous FM responses at fixed values of the structural selectivity parameter G. For each G, the dashed line denotes the deterministic EM trend, the solid line denotes the FM ensemble mean, and the shaded band denotes the F M interquartile interval $q_{10}$–$q_{90}$. (**B**) reports the corresponding FM bandwidth, expressed as $q_{90}-q_{10}$, as a function of χ. The color code is the same in both panels: blue for G=0.20, orange for G=0.50, green for G=0.80, and red for G=0.95.

The mean-field comparison in Figure 4 shows that pore-size heterogeneity preserves the main organisation of the coherence landscape while introducing local deviations in the ensemble-averaged response. A complementary question is how broadly the heterogeneous response is distributed around that mean trend. Figure 5 addresses this point by comparing the deterministic EM sectional profiles of normalised coherence with the corresponding FM ensemble mean and with the interquantile envelope generated by Monte Carlo pore populations.

The sectional representation shows how the heterogeneous FM response is distributed around the deterministic EM trend at fixed structural selectivity. In Panel A, the solid FM mean curves remain close to the corresponding dashed EM profiles for all reported values of G, indicating that pore-size heterogeneity does not replace the deterministic sectional trend with a different trajectory. Its main effect is instead to generate a finite response envelope around that trend, represented by the shaded $q_{10}$–$q_{90}$ band.

The amplitude of this envelope depends strongly on G. At low G, the FM band remains narrow over the entire χ range, showing that when defect-mediated pathways still contribute substantially to the transport state, selective-population heterogeneity has only a limited effect on the accessible coherence response. As G increases, the band becomes progressively wider, indicating that the influence of pore-size dispersion is amplified when the membrane response is more strongly controlled by the selective branch. Under the present parameterisation, the FM average also lies systematically above the dashed EM trend, so that heterogeneity produces not only broadening but also a modest upward displacement of the average normalised coherence.

Panel B expresses the same result directly in terms of bandwidth. The quantity $q_{90}-q_{10}$ increases with G throughout the domain, with the strongest broadening observed for the highest-G profile and the weakest for the lowest-G one. The width also increases along χ, particularly in the high-G regime. In practical terms, this means that nanochemical activation enlarges the interval of heterogeneous responses most clearly when the transport state is already strongly selective.

Beyond the analysis of Monte Carlo-generated pore populations, it is important to consider how model predictions might evolve under alternative structural scenarios. For example, varying the pore-size distribution or introducing additional parameters such as channel connectivity and tortuosity could further refine the transport response. These factors may play a significant role in real membrane systems, where non-uniformity and complex architecture contribute to overall performance.

Moreover, future studies could incorporate dynamic changes in pore structure caused by environmental influences, chemical modifications, or operational stresses. Such adaptations may alter the selective population over time, affecting both water and solute transport mechanisms. A comprehensive understanding of these effects will enhance the predictive power of FM approaches and support the optimisation of advanced membrane designs.

Taken together, Figure 5 shows that the principal contribution of FM at the sectional level is to broaden the accessible coherence response around the deterministic EM trend while preserving the same overall trajectory. The broadening remains limited in weakly selective states and becomes progressively more pronounced as the system moves toward the high-G regime.

### 3.3. Influence of Experimentally Derived Pore Size Distributions

#### 3.3.1. Experimentally Derived Pore-Size Distribution

The experimentally derived pore-size distribution provides the structural input used to replace the synthetic selective population adopted in the Monte Carlo analysis. Whereas Section 3.2 isolated the effect of controlled heterogeneity under an idealised pore ensemble, the present analysis examines whether the same transport organisation is retained when the selective population is instead derived from an experimentally reported pore-size distribution representative of nanostructured forward-osmosis membranes.

**Figure 6:**
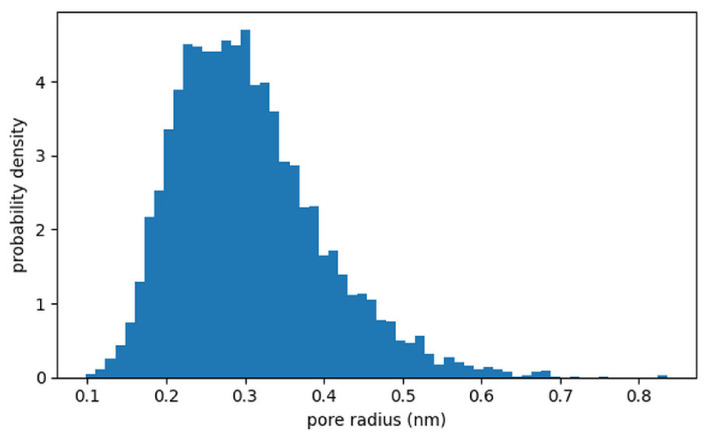
Experimentally derived pore-size distribution used in the heterogeneous full model. Probability-density histogram of pore radii adopted as input for the experimentally informed FM simulations. The distribution is characterized by a dominant sub-nanometric population associated with selective transport and by a lower-probability tail extending toward larger pores, which is expected to disproportionately affect solute leakage.

Figure 6 shows the experimentally derived pore-size distribution used in the heterogeneous FM. The imported distribution is asymmetric, with a dominant sub-nanometric population concentrated in the selective range and a lower-probability tail extending toward larger pore radii. This structure is relevant since the narrow-pore population sustains selective transport, whereas the less populated large-radius tail can contribute disproportionately to reverse solute leakage even when its statistical weight remains limited.

The experimentally informed distribution, therefore, provides a more physically constrained test of the FM framework than the synthetic Monte Carlo ensemble alone. Rather than asking only how generic heterogeneity broadens the deterministic EM response, the analysis now evaluates how an asymmetric pore population representative of experimentally reported nanostructured membranes redistributes the transport response within the same framework.

#### 3.3.2. Transport-Field Response in the High-Selectivity Regime

**Figure 7:**
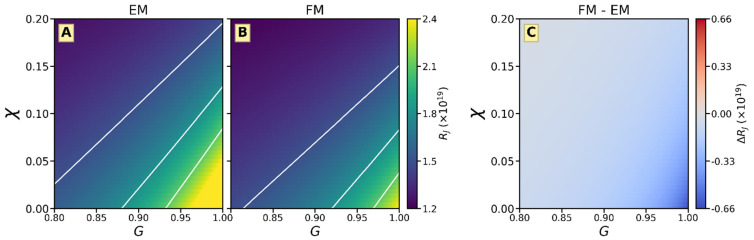
Transport landscape from experimentally derived pore-size distribution. Comparison between EM (**A**) and FM (**B**) driven by the experimental pore-size distribution shown in Figure 6, restricted to $G\in \left[0.8,1.0\right]$ and $\chi \in \left[0,0.2\right]$. (**A**,**B**) are shown on the same linear scale, $R_{J}=1.2$ to $2.4\times 10^{19}$, so that the local redistribution induced by experimental pore heterogeneity can be compared directly within the same selective-dominant transport window. (**C**) shows the difference field ${R_{J}}_{FM}-{R_{J}}_{EM}$.

The effect of experimentally informed heterogeneity becomes most visible when the analysis is restricted to the selective-dominant regime. Figure 7, therefore, compares the deterministic EM with the FM driven by the experimental pore-size distribution of Figure 6 within the restricted domain G∈[0.8,1.0] and χ∈[0,0.2]. Panels A and B are shown on the same linear scale, $R_{J}=1.2$ to $2.4\times 10^{19}$, so that the local redistribution induced by experimental pore heterogeneity can be compared directly within the same transport window. Panel C reports the signed difference field ${R_{J}}_{FM}-{R_{J}}_{EM}$.

Within this restricted domain, the main directional organisation is preserved across the two formulations. In both EM and FM, $R_{J}$ increases with increasing G and decreases with increasing χ, so that the strongest responses remain localised toward the lower-right sector of the state-space. The experimentally informed FM solution therefore preserves the first-order transport ordering already present in the deterministic EM reference, while resolving how a realistic asymmetric pore population redistributes the local response in the high-selectivity regime.

This directional consistency is confirmed by the signed gradient variables. The mean derivatives remain positive with respect to G and negative with respect to χ in both descriptions, with $\langle \partial R_{J}/\partial G\rangle_{EM}=7.33\times 10^{18}$, $\langle \partial R_{J}/\partial \chi \rangle_{EM}=-9.60\times 10^{18}$, $\langle \partial R_{J}/\partial G\rangle_{FM}=3.93\times 10^{18}$, and $\langle \partial R_{J}/\partial \chi \rangle_{FM}=-7.37\times 10^{18}$. These values show that experimental heterogeneity does not invert the transport gradients; rather, it weakens their average magnitude, indicating that the FM field is less sensitive than EM to both structural selectivity and nanochemical perturbation within this domain.

At the same time, the FM field lies below the EM field over most of the displayed window. This is shown directly in Panel C, where ${R_{J}}_{FM}-{R_{J}}_{EM}$ is predominantly negative and becomes more negative toward the lower-right sector, that is, precisely where the selective contribution is strongest. The experimentally derived pore ensemble, therefore, does not introduce a random perturbation of the deterministic field. Instead, it produces a coherent downward redistribution of the response relative to the EM reference.

In physical terms, this behaviour indicates that the asymmetric experimental pore population depresses the effective selective-branch response while preserving the same global dependence on G and χ. The role of experimentally informed FM is therefore not to reorganize the topology of the transport field, but to quantify how a realistic pore-size distribution lowers the selective-branch response relative to the deterministic reference while maintaining the same directional transport structure. In this sense, Figure 7 provides a more specific conclusion than the synthetic Monte Carlo analysis alone: in the high-selectivity regime, realistic heterogeneity preserves the monotonic transport ordering imposed by EM, but shifts the local response downward and reduces gradient intensity.

### 3.4. Implications of Structural Heterogeneity for Transport Coherence

The BM–EM–FM results define a consistent hierarchy in the mechanisms governing transport coherence in heterogeneous FO membranes. In BM, redistribution between selective and defect-mediated pathways is controlled primarily by the structural parameter G, and the coherence response evolves relatively smoothly because chemistry and geometry remain effectively separable. EM introduces the first genuine reorganisation of this reference state. Once the selective radius and the effective weight of the selective population become functions of the nanochemical state χ, the coupled response of water and solute transport becomes intrinsically asymmetric, and the coherence landscape acquires a more strongly state-dependent structure, especially where selective pathways dominate the conductive network.

FM extends this picture without replacing it. Structural heterogeneity acts mainly by redistributing the deterministic EM response through selective-population dispersion. In the Monte Carlo implementation, this appears primarily as broadening around the EM trend; in the experimentally informed implementation, the same directional organisation is preserved while the local response is shifted downward in the high-selectivity regime. Pore-size heterogeneity, therefore, modulates the amplitude and spread of the coherence response rather than introducing an independent transport logic.

Within this hierarchy, the ratio $R_{J}=J_{s}/J_{w}$ provides a compact description of how transport progressively shifts from a state governed predominantly by selective pathways to one increasingly influenced by weakly selective or defect-mediated routes. Transport coherence is therefore best interpreted not as ideal selectivity or abrupt functional collapse, but as the persistence, and eventual loss, of a common effective transport organisation for water permeation and solute leakage within a heterogeneous membrane network.

**Figure 8:**
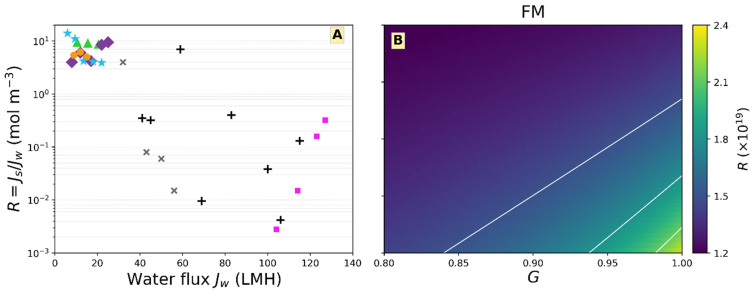
Experimental transport states and FM coherence landscape. (**A**) Experimental points in the $\left(J_{w},R_{J}\right)$ plane, with $R_{J}$ expressed in mol m^−3^, obtained by converting the experimental $J_{s}/J_{w}$ ratio from g L^−1^ using the NaCl molar mass. Green triangles: Phillip et al. [33] (HTI-CTA); purple diamonds: Rufuss et al. [34] (biomimetic HFFO); orange circles: Nasr et al. [35] (CTA salinity sweep); magenta squares: Nasr et al. [35] (SG3 salinity sweep); black plus symbols: Nasr et al. membrane-state series at 215 μm; dark-gray x symbols: Nasr et al. membrane-state series at 265 μm [35]; cyan stars: TFN-GO membranes from Idris et al. [36]. (**B**) FM transport landscape in the internal state-space (G,χ), computed from the parameters in Table 1 and the processed pore-size distribution. The colour scale reports $R_{J}$(×10^19^). This panel provides a structural-coherence landscape in internal model coordinates and is compared with Panel A at the level of ordered transport regimes rather than by direct axis equivalence. White isolines correspond to the same contour levels adopted in Figure 7B (1.5, 1.8, 2.1 × 10^19^). (**C**) Exploratory FM landscape incorporating external and internal concentration polarisation at *k*_ECP_ = 3.5 × 10^−5^ m s^−1^ and *k*_ICP_ = 4.5 × 10^−5^ m s^−1^. The colour scale reports $R_{J}$ for both (**B**) and (**C**) and white isolines denote the same contour levels used in Figure 7B (1.5, 1.8, 2.1 × 10^19^) for both panels (**B**,**C**).

The magnitude of the BM–EM–FM differences is moderate in parts of the state-space, especially at low G, but this behaviour is itself mechanistically informative: it indicates that coherence loss becomes detectable mainly when the selective pathway population dominates the transport response. The framework is therefore intended to diagnose regime-dependent reorganisation rather than to amplify small numerical differences into universal performance claims. The quantitative impact of these device-level effects on the inter-model hierarchy is examined through Figure 8C, which incorporates explicit ECP and ICP corrections within the FM framework.

## 4. Discussion

### 4.1. Interpretation of Transport Coherence and Diagnostic Role of $R_{J}$ Across the BM–EM–FM Hierarchy

The BM–EM–FM hierarchy provides a consistent interpretation of how transport coherence is progressively lost in heterogeneous FO membranes. Within the present framework, BM defines the reference state in which selective and defect-mediated pathways coexist under fixed selective geometry, so that the transport response remains governed primarily by the structural selectivity parameter G. This baseline is important because it isolates pathway competition before explicit chemistry-dependent accessibility loss is introduced. In this regime, the coherence landscape evolves relatively smoothly, and the dominant gradients remain largely aligned with the structural coordinate. Thus, we remark that transport coherence is not introduced here as a universal thermodynamic quantity, but as an operational descriptor of whether water and solute transport remain governed by a common effective pathway organisation.

EM introduces the first genuine reorganisation of that reference state. Once the effective selective radius and the functional contribution of the selective branch become dependent on the nanochemical state χ, chemistry no longer acts as a secondary modulation but becomes a state-organising variable. The principal EM result is therefore not a generic reduction of transport, but a redistribution of the balance between selective permeation and leakage in the high-G region, where the membrane response is most strongly controlled by the selective pathway population. In physical terms, chemistry–geometry coupling reshapes the coherence landscape because water permeation and solute leakage no longer respond symmetrically to the same selective environment.

FM extends this interpretation without replacing it. In the Monte Carlo implementation, pore-size heterogeneity broadens the response around the deterministic EM trend while preserving the main topology of the field. In the experimentally informed implementation, the literature-derived pore-size distribution preserves the same directional organisation but redistributes the local response downward relative to EM in the high-selectivity domain. The role of FM is therefore to quantify how structural heterogeneity modulates an already established deterministic organisation rather than to introduce an independent transport logic. Coherence loss thus arises first from pathway competition and chemistry-dependent accessibility, whereas heterogeneity mainly controls how strongly and how broadly that redistribution is expressed. Figure 8C shows that concentration polarisation does not merely rescale this picture: it selectively amplifies the inter-model differences, with the BM–FM coherence gap at χ = 0.40 growing from 0.21 without CP to 0.76 with ECP + ICP. This amplification occurs because CP reduces $J_{w}$ most strongly where the selective branch is most active, compressing the effective selective contribution precisely where chemistry–geometry coupling and pore-size heterogeneity are most diagnostic. The moderate inter-model differences noted in parts of the pathway-level analysis, therefore, represent a lower bound on the diagnostic contrast available under realistic operating conditions.

Within this hierarchy, the ratio $R_{J}=J_{s}/J_{w}$ functions as a diagnostic descriptor of transport organisation. Its value lies in revealing whether water permeation and reverse solute leakage remain governed predominantly by the same selective pathway population or are progressively redistributed toward different effective routes. Low and smoothly varying values of $R_{J}$ identify transport states in which the selective network remains functionally dominant, whereas increasing values indicate progressively stronger leakage-prone contributions relative to the hydraulic response. The interpretive value of $R_{J}$ is therefore not that it replaces the broader set of descriptors required for full membrane characterization, but that it condenses pathway competition and transport reorganisation into an experimentally accessible quantity.

### 4.2. Relation to Heterogeneous Membrane Structure and Experimental Observations

Figure 8 compares one experimental observable space with two model-based internal landscapes. Panel A reports the experimental datasets in the observable plane ($J_{w},R_{J}$), where $J_{w}$ is the measured water flux and $R_{J}=J_{s}/J_{w}$ is the experimentally accessible coherence descriptor. Panel B reports the FM response in the internal state-space (G,χ), where G represents the effective dominance of the selective pathway population and χ the nanochemical condition modulating pathway accessibility. Panel C extends the same FM internal landscape by incorporating a bounded concentration-polarisation perturbation based on external and internal boundary-layer attenuation terms, using the standard exponential relations [31,32]:

(22) $$C_{ECP}=C_{bulk}exp\left(-\frac{J_{w}}{k_{ECP}}\right),C_{ICP}=C_{ECP}exp\left(-\frac{J_{w}}{k_{ICP}}\right),$$

evaluated at the same internal states (G,χ). The resulting field is shown on the same absolute color scale as panel B to highlight the concentration-induced perturbation of the FM coherence landscape. In the executable representation used for panel C, the hydraulic FM proxy was scaled to $J_{w,max}=115$ LMH, and the concentration-polarisation terms were evaluated using $k_{D}=3.5\times 10^{-5}$ m s^−1^, $k_{F}=4.5\times 10^{-5}$ m s^−1^, $C_{D}=1.0$, $C_{F}=0.05$, and a near-symmetric solute attenuation exponent m=0.975. This parameterisation represents a mild concentration-polarisation regime in which the water-driving term and the reverse-solute term are affected on nearly the same boundary-layer scale.

The three panels are compared at the level of transport ordering: whether experimental membrane states in Panel A occupy high- or low-leakage regimes broadly consistent with the coherence organisation described by Panels B and C, rather than by direct axis equivalence. The relation among the panels is therefore mechanistic and ordinal rather than coordinate-by-coordinate. In particular, $J_{w}$ is not equivalent to G: the former is an observable transport output, whereas the latter is an internal structural descriptor of the model.

Under this distinction, the experimental topology in Figure 8A remains highly informative. The datasets occupy the ($J_{w},R_{J}$) plane in an ordered rather than random manner. Low-flux states are associated with relatively high values of $R_{J}$, intermediate states define a transition region in which $R_{J}$ decreases as $J_{w}$ increases, and high-flux states occupy a low-$R_{J}$ domain in which reverse solute leakage is strongly suppressed relative to water transport. This membrane-state progression is important because it traces a continuous displacement across the observable plane, supporting the interpretation that real membranes may operate in more coherent or more leakage-prone transport regimes rather than forming an undifferentiated cloud of responses.

The FM landscape in Figure 8B provides the structural interpretation of that ordering at the level intended by the model. Low values of the FM descriptor correspond to states in which water permeation and solute leakage remain more tightly governed by the selective pathway population, whereas higher values identify states in which leakage-prone pathways contribute more strongly to the observable response. The theoretical field should therefore be compared with the experimental data as a correspondence between regimes. Under this reading, the low-$J_{w}$, high-$R_{J}$ experimental cluster is consistent with less coherent sectors of the FM landscape; the intermediate states are consistent with a redistribution zone; and the high-$J_{w}$, low-$R_{J}$ states are consistent with sectors in which the selective network remains functionally dominant.

Panel C shows that concentration polarisation introduces a limited but spatially structured perturbation of the FM coherence landscape. Because the correction is evaluated from the hydraulic FM proxy, its effect is more visible in the hydraulically stronger sector of the internal state-space, while the same absolute color scale allows direct comparison with panel B. The perturbation does not invert the map or displace the principal high- and low-$R_{J}$ domains: low values remain localised toward lower G and higher χ, whereas the highest values remain associated with high G and low χ. This behaviour supports the interpretation that moderate concentration polarisation can modify the apparent coherence level while preserving the dominant FM ordering.

Within this regime-based reading, the graphene- and GO-containing membrane families in Figure 8A are especially informative because they cluster toward experimentally lower- $R_{J}$ states. This placement is consistent with transport regimes in which structural modification improves hydraulic productivity while reducing the relative contribution of leakage-prone pathways. In FM terms, these membrane families are therefore associated with sectors more strongly governed by selective nanochannels and less influenced by defect-mediated leakage, without implying a one-to-one assignment of the experimental points to unique (G,χ) coordinates.

Figure 8 should therefore be read as a structured theory–experiment comparison at the level of ordered transport regimes. Panel A shows how real membranes populate the observable plane, panel B shows the internal coherence landscape described by the FM framework, and panel C shows the corresponding landscape after a mild concentration-polarisation perturbation. Their qualitative correspondence supports the physical plausibility of the BM–EM–FM interpretation and its usefulness for organising transport states, rather than membrane-specific validation in a strict predictive sense. Since Figure 8A compiles literature data obtained from different membrane families and operating conditions, the comparison is interpreted as a qualitative transport-ordering map after unit harmonisation, rather than as a strict quantitative cross-study performance comparison.

## 5. Conclusions

This work introduced a regime-based diagnostic framework for analysing transport coherence in heterogeneous forward osmosis membranes. The BM–EM–FM hierarchy was used to determine when water permeation and reverse solute leakage remain governed predominantly by the same selective pathway population, and when they progressively decouple as transport is redistributed toward weakly selective or defect-mediated routes.

Within this framework, the ratio $R_{J}=J_{s}/J_{w}$ is used as a compact descriptor of transport coupling and of the extent to which water permeation and solute leakage remain governed by a common effective transport organisation.

BM defines the reference state in which transport coherence evolves smoothly because selective geometry remains fixed and chemistry acts only through interfacial transport factors. EM introduces the first genuine reorganisation of this landscape: once nanochemical activation reduces both the effective selective radius and the functional contribution of the selective pathway population, chemistry–geometry coupling becomes a state-organizing mechanism rather than a simple modulation. FM extends this picture by incorporating pore-size heterogeneity within the selective population. In the Monte Carlo implementation, heterogeneity broadens the deterministic EM response while preserving its main topology. In the experimentally informed implementation, realistic pore-size asymmetry preserves the same directional ordering but shifts the local response and reduces gradient intensity in the high-selectivity regime. The concentration-polarisation extension of the FM landscape further shows that moderate external and internal boundary-layer effects introduce a spatially structured perturbation without changing the dominant coherence topology. In this sense, concentration polarisation modifies the apparent coherence level under operating conditions, while the underlying BM–EM–FM ordering remains interpretable.

Transport coherence is therefore interpreted as the persistence of a common effective transport organisation for water permeation and solute leakage within a heterogeneous pathway network. Its loss corresponds to a progressive redistribution of transport toward increasingly leakage-prone routes. Under this interpretation, $R_{J}$ is most valuable as an experimentally accessible descriptor of pathway-level transport organisation and of the progressive onset of leakage-prone transport.

Future work should incorporate additional device-level effects such as support-layer resistance, fouling, compaction, and membrane aging, and may explore physics-informed parameter inference to connect internal transport landscapes with membrane-specific operating behaviour.

## Data Availability

The original contributions presented in this study are included in the article/Supplementary Materials. Further inquiries can be directed to the corresponding author.

## Supplementary Materials

The following supporting information can be downloaded at: https://www.mdpi.com/article/10.3390/membranes16060211/s1, Figure S1: Robustness-oriented three-panel analysis of the BM–EM–FM framework. (A) Primary robustness analysis of α, β, and γ, shown through sectional profiles of ${\tilde{R}}_{EM}(\chi )$ at G=0.80. (B) FM heterogeneity sensitivity shown through sectional profiles of ${\tilde{R}}_{FM}(\chi )$ at G=0.80 for the reference, widened, and heavy-tail perturbations of the reference selective pore-size distribution; the inset highlights the high-χ range. (C) Secondary sensitivity of the FM coherence response to transport lengths $L_{s},L_{d}$ and slip lengths $b_{s},b_{d}$, quantified as the percent shift in mean ${\tilde{R}}_{FM}$ over the high-selectivity window G∈[0.8,1.0], χ∈[0,0.2] under 0.5×, 1×, and 2× perturbations; Figure S2: Maps of the coherence ratio $R=J_{s}/J_{w}$ in the $\left(G,\chi \right)$ state space for the BM, EM, and FM formulations. The markers P1 and P2 denote representative checkpoints used as visual consistency tests: the local color at each point is qualitatively consistent with the corresponding value indicated by the color bar. White lines represent selected iso- R contours; Figure S3: Extended model (EM) map of the coherence ratio $R=J_{s}/J_{w}$ as a function of structural selectivity G and nanochemical state χ. Points P1 and P2 indicate representative checkpoints used to verify that the local field color is qualitatively consistent with the corresponding value read from the color bar. White lines denote selected iso-R contours; Figure S4: FM map of the coherence ratio $R=J_{s}/J_{w}$ as a function of G and χ. The points P1 and P2 are included as in Figures S2 and S3 as representative checkpoints to verify that the local map color is qualitatively consistent with the associated color-bar value. White lines indicate selected iso-R contours; Table S1: Experimental coherence ratios after unit harmonisation (from Nasr et al.’s dataset [1]); Table S2: Parameter classes and robustness test roles. The table reports the parameter groups considered in the executable robustness analysis of primary framework coefficients (α, β, and γ); FM heterogeneity perturbations are applied to the reference selective pore-size distribution and secondary transport-scale sensitivities ($L_{s}$, $L_{d}$, $b_{s}$, and $b_{d}$). The listed perturbations define the reference robustness tests used to assess the stability of the qualitative BM–EM–FM interpretation. References [9,15,18,20,21,22,24,25,35,37,38,39] are cited in the Supplementary Materials.
