# Supplementary material for: Transport Coherence Loss in Heterogeneous Forward Osmosis Membranes: A Hierarchical Diagnostic Framework
Source: Membranes (Basel). 2026 Jun 18;16(6):211. doi: 10.3390/membranes16060211 (PMC13304315; doi:10.3390/membranes16060211)
Supplement: Supplementary file 1 [file membranes-16-00211-s001.zip › membranes-4288409-supplementary.pdf]

membranes

MDPI

Supplementary Materials

Transport Coherence Loss in Heterogeneous Forward Osmosis Membranes: A Hierarchical Diagnostic Framework

Maurizio Viviani [1], Nicola Bragazzi [2], Gaositwe Bolani [3], Simonetta Papa [4], Luca Giacomelli [4],* and Roberto Eggenhöffner [4]

[1] Robotics.it LLC, 9649 West Olympic Blvd, Suite 7, Beverly Hills, CA 90212, USA; digimatronics@gmail.com
[2] Laboratory for Industrial and Applied Mathematics (LIAM), Department of Mathematics and Statistics, York University, Toronto, ON M3J 1P3, Canada; robertobragazzi@gmail.com
[3] Clinical Pathology, School of Pathology, Faculty of Health Sciences, University of the Witwatersrand, Johannesburg, South Africa; gaosib@gmail.com
[4] Polistudium SrL, VIA SOLFERINO 7, 20121 Milano, Italy; simonetta.papa@polistudium.it (S.P.); roberto.eggenhoffner@gmail.com (R.E.)
* Correspondence: luca.giacomelli@polistudium.it

1. Pore-Network Architecture and Chemistry-Dependent Control Parameters

1.1. Unit harmonization and experimental coherence ratios from the Nasr dataset

To assess whether the coherence diagnostics derived from the FM framework remain qualitatively consistent with experimentally observed FO behavior, we analyzed the experimental data reported by Nasr et al. [1] for PES/GO mixed-matrix forward osmosis membranes, as shown in Table S1. These experiments are particularly suitable for this comparison because they report both water flux and reverse solute flux under controlled operating perturbations.

$$R = \frac{J_s}{J_w}, \quad (1)$$

The above is used here as an interpretable indicator of the relative coupling between water permeation and reverse-solute leakage.

Because the Nasr dataset reports J_w in LMH and J_s in GMH, the raw ratio J_s/J_w has units of $g\,L^{-1}$. To express the data in the same units adopted in the FM framework, the ratio was converted into molar concentration units according to the conversion factor $\frac{1000}{M_{\mathrm{NaCl}}}\left(\frac{J_s}{J_w}\right)$, where $M_{NaCl} = 58.44\ g\,/mole$. Thus, after the conversion, R is presented in units of $\mathrm{mol\ m^{-3}}$.

The purpose of this supplementary analysis is not to provide a point-by-point validation of the BM–EM–FM model, but to document how the experimentally reported flux ratios were harmonized and how the resulting values map onto the coherence regimes discussed in the main text. Consistently with the manuscript scope, the experimental comparison is used as an external qualitative consistency check and transport-ordering test, not as a membrane-specific predictive validation.

Table S1. Experimental coherence ratios after unit harmonization (from Nasr et al.'s dataset [1]).

Membrane	J_w (LMH)	J_s (GMH)	J_s/J_w (g L^{-1})	R (mol m^{-3})
PES bare	60	25.9	0.432	7.39
SG1	83	2.00	0.024	0.411
SG2	99.7	0.22	0.0022	0.0376
SG3	115	0.03	0.00026	0.00445

 https://doi.org/10.3390/membranes16060211

Experimental water-flux and reverse-solute-flux data from the Nasr dataset [1] after conversion to units. The converted quantity is the coherence descriptor used throughout the present study. The table documents the unit harmonization step underlying the comparison with the FM coherence landscape.

The converted Nasr data provide an experimental consistency check for the coherence response regions identified by the FM framework. In particular, SG2 and SG3 occupy a low-R range spanning approximately 10^{-3} to $10^{-1}\ mol\, m^{-3}$, whereas the bare PES membrane remains above 7 $mol\, m^{-3}$. This separation indicates that membranes with comparable or even improved water productivity may correspond to markedly different internal transport states when examined through the coherence descriptor. In the language of the present model, low-R states are consistent with transport remaining more strongly governed by the selective pathway population, whereas high-R states indicate progressively stronger contribution from non-selective or defect-mediated routes. This is the central reason why R is informative: it captures internal transport redistribution that is not fully visible from inspection of J_w alone.

The key outcome of this comparison is therefore not the prediction of absolute flux magnitudes, but the qualitative reproduction of experimentally observed transport reordering. In this sense, the Nasr dataset supports the interpretation advanced in the main text: water flux may remain relatively high while the solute-to-water flux ratio changes markedly, signaling progressive loss of transport coherence as leakage-prone pathways become functionally more relevant.

2. Parameter robustness and executable sensitivity tests for the BM–EM–FM figure family

2.1. Robustness strategy and perturbed quantities

The transport maps reported in Figures 2–7 were generated from the executable BM–EM–FM framework using the reference working parameter set listed in Table 1 of the main manuscript. The purpose of the present appendix is to verify whether the principal mechanistic conclusions remain qualitatively stable under systematic perturbation of that reference set, without introducing a new model layer or attempting membrane-specific refitting.

The robustness analysis is organized around three parameter classes. First, physically grounded reference inputs such as r_s, r_d, a, and D_0 and, in the experimentally informed FM implementation, the reference selective pore-size distribution define the physical baseline of the executable regime and are not treated as freely re-estimated coefficients in the primary tests. Second, the transport lengths L_s and L_d and slip lengths b_s and b_d are treated through secondary sensitivity tests in order to verify that the BM–EM–FM interpretation does not depend strongly on the chosen executable transport scales. Third, the framework coefficients α, β, and γ are treated as the primary robustness parameters because they control how chemistry-dependent exclusion, selective radius contraction, and loss of selective accessibility deform the transport and coherence landscapes.

The driving terms Δp_{eff} and Δc are interpreted as executable reference scales for the water and solute branches rather than as one-to-one representations of a unique operating condition. Since Δp_{eff} enters only J_w and Δc enters only J_s, a joint proportional rescaling changes the absolute flux magnitudes but leaves $R = J_s/J_w$ and $\tilde{R}$ unchanged. For this reason, the robustness analysis reported here is centered on α, β, and γ; on secondary perturbations of L_s, L_d, b_s, and b_d; and, in FM, on the width and tail perturbations of the reference selective pore-size distribution.

Across all perturbed cases, robustness is evaluated through the same diagnostics used in the main text: the topology of the transport and coherence fields, the localization

of the EM-induced coherence reorganization in the high-G regime, the persistence of FM broadening and redistribution around the deterministic trend, and the preservation of the interpretation of R as a descriptor of the transition from selective-dominant to leakage-prone transport states.

2.2. Interpretation of Table S2 and Figure S1

Table S2 summarizes how each parameter group is treated within the robustness analysis. The table distinguishes between secondary executable transport scales (L_s, L_d, b_s, and b_d), primary framework coefficients (α, β, and γ), and the FM heterogeneity input represented through perturbations of the reference selective pore-size distribution. Its function is to identify which quantities are structurally consequential, which are treated as secondary sensitivity variables, and how each class is perturbed in the executable tests.

Table S2. Parameter classes and robustness test roles. The table reports the parameter groups considered in the executable robustness analysis of primary framework coefficients (α, β, and γ); FM heterogeneity perturbations are applied to the reference selective pore-size distribution and secondary transport-scale sensitivities (L_s, L_d, b_s, and b_d). The listed perturbations define the reference robustness tests used to assess the stability of the qualitative BM–EM–FM interpretation.

Parameter/ group	Role in the model	Status in robustness analysis	Suggested perturbation
L_s, L_d	Transport lengths	Secondary sensitivity	0.5×, 1×, 2×
b_s, b_d	Slip lengths	Secondary sensitivity	0.5×, 1×, 2×
α	Electrostatic attenuation	Primary robustness parameter	Weak/ref/strong
β	Chemistry–geometry contraction	Primary robustness parameter	Zero to strong
γ	Selective accessibility loss	Primary robustness parameter	Zero to strong
$p(r)$	FM heterogeneity input	Varied through width/tail tests	Reference/widened/heavy-tail

Figure S1 reports the corresponding robustness results in a compact graphical form. Panel A shows that the primary framework coefficients modify the sectional coherence response $\tilde{R}_{FM}(\chi)$ at fixed $G = 0.80$, with γ producing the strongest deformation because it acts directly on the effective weight of the selective pathway population. Panel B shows the effect of FM heterogeneity perturbations on the sectional response $\tilde{R}_{FM}(\chi)$ at the same G, comparing the reference case with widened and heavy-tail perturbations of the reference selective pore-size distribution. The three curves preserve the same overall monotonic organization, while the inset highlights the small but systematic differences that emerge in the high- χ range. Panel C shows that perturbations of L_s, L_d, b_s, and b_d mainly produce secondary shifts in the mean normalized coherence response, indicating that the principal FM interpretation is not controlled by the chosen transport scales alone.

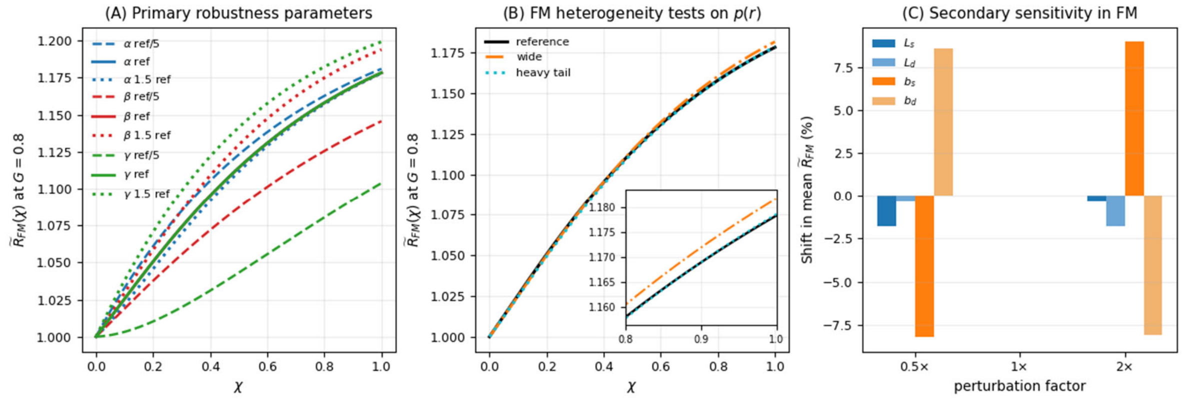

Figure S1. Robustness-oriented three-panel analysis of the BM–EM–FM framework**.** (A) Primary robustness analysis of α, β, and γ, shown through sectional profiles of $\widetilde{R}_{EM}(\chi)$ at G = 0.80. (B) FM heterogeneity sensitivity shown through sectional profiles of $\widetilde{R}_{FM}(\chi)$ at G = 0.80 for the reference, widened, and heavy-tail perturbations of the reference selective pore-size distribution; the inset highlights the high- χ range. (C) Secondary sensitivity of the FM coherence response to transport lengths L_s, L_d and slip lengths b_s, b_d, quantified as the percent shift in mean $\widetilde{R}_{FM}$ over the high-selectivity window G ∈ [0.8,1.0], χ ∈ [0,0.2] under 0.5 ×, 1 ×, and 2 × perturbations.

Taken together, Table S2 and Figure S1 support the same conclusion: the most consequential robustness directions are associated with chemistry-dependent accessibility loss and with the structure of the FM heterogeneity input, whereas transport lengths and slip lengths play a secondary role. The qualitative BM–EM–FM hierarchy, therefore, remains stable under substantial executable perturbation of the reference set.

3 Step-by-step numerical reconstruction of representative BM–EM–FM states and verification against Figures 1–7 and 8B

3.1. Outline of the procedure

This appendix provides an explicit numerical reconstruction of representative BM, EM, and FM transport states. The purpose is to document, in a transparent and reproducible form, how the working parameter set generates the values plotted in the transport maps and in the restricted high-selectivity windows used in the main text. The appendix follows the same transport architecture introduced in Section 2 of the manuscript and does not introduce an additional model layer.

Two classes of checkpoints are used. The first class consists of panel-specific local verification points, denoted by P_1^{BM}, P_1^{EM}, and P_1^{FM}, chosen within the most informative visible region of each corresponding map. The second class consists of a common comparison point P_2, shared across BM, EM, and FM and used for direct cross-model comparison. The adopted coordinates are

$$P_1^{BM} = (0.650,0.200), P_1^{EM} = (0.920,0.050), P_1^{FM} = (0.950,0.050), \quad (2)$$

and

$$P_2 = (0.985,0.015). \quad (3)$$

The panel-specific P_1 points are used only as local consistency checks inside the visible plotting windows and are not intended for one-to-one cross-model comparison. By contrast, P_2 is the common coordinate used to compare BM, EM, and FM directly. These choices are consistent with the revised figure windows, namely $0.50 \le G \le 1.00$, $0.00 \le \chi \le 0.40$ for BM, and $0.80 \le G \le 1.00$, $0.00 \le \chi \le 0.20$ for EM and FM.

The pathway-scale water and solute expressions are written in explicit, calculable form as

$$J_w^{(\text{path})} = n_p \left(\frac{\pi r^4}{8\mu L}\right)\left(1+\frac{4b}{r}\right)\Delta p_{\text{eff}}, \tag{4}$$

$$J_s^{(\text{path})} = n_p \left(\frac{\pi r^2 D_0}{L}\right) K_{\text{steric}}(\lambda)\, K_{\text{elec}}(\chi)\, \Delta c, \tag{5}$$

with

$$\lambda = \frac{a}{r}, K_{\text{steric}}(\lambda) = \max\,(1-\lambda, 0)^2, K_{\text{elec}}(\chi) = \exp\,(-\alpha\chi). \tag{6}$$

The working parameter set is taken consistently with Table 1 of the main manuscript:

$$r_s = 0.500\ \text{nm}, r_d = 1.500\ \text{nm}, \tag{7}$$

$$L_s = L_d = 100\ \text{nm}, b_s = 20.0\ \text{nm}, b_d = 10.0\ \text{nm}, \tag{8}$$

$$a = 0.325\ \text{nm}, \alpha = 2.20, \beta = 0.20, \gamma = 0.35, \tag{9}$$

$$D_0 = 1.60 \times 10^{-9}\ \text{m}^2\ \text{s}^{-1}, \Delta c = 100\ \text{mol m}^{-3}, \Delta p_{\text{eff}} = 1.00 \times 10^5\ \text{Pa}. \tag{10}$$

3.2. BM reconstruction at P_1^{BM} and P_2

This section reconstructs the baseline model. In BM, the selective radius is kept fixed at $r_s = 0.50$ nm, so chemistry does not contract the selective geometry. The chemistry dependence enters only through the electrostatic attenuation of the selective solute contribution.

The fixed selective steric ratio is

$$\lambda_s^{BM} = \frac{a}{r_s} = \frac{0.325}{0.500} = 0.650, \tag{11}$$

which gives

$$K_{\text{steric},s}^{BM} = (1-0.650)^2 = 0.122. \tag{12}$$

The fixed selective hydraulic term is

$$q_{w,s}^{BM} = \left(\frac{\pi r_s^4}{8\mu L_s}\right)\left(1+\frac{4b_s}{r_s}\right)\Delta p_{\text{eff}} = 3.95 \times 10^{-22}. \tag{13}$$

The defect hydraulic and solute terms are

$$q_{w,d} = \left(\frac{\pi r_d^4}{8\mu L_d}\right)\left(1+\frac{4b_d}{r_d}\right)\Delta p_{\text{eff}} = 5.50 \times 10^{-21}, \tag{14}$$

$$q_{s,d} = \left(\frac{\pi r_d^2 D_0}{L_d}\right)\Delta c = 1.13 \times 10^{-18}. \tag{15}$$

For $P_1^{BM} = (0.650, 0.200)$, the electrostatic factor is

$$K_{\text{elec}}(0.200) = \exp(-2.20 \times 0.200) = \exp(-0.440) = 0.644. \tag{16}$$

Hence, the selective solute term becomes

$$q_{s,s}^{BM}(P_1) = 9.91 \times 10^{-21}. \tag{17}$$

The BM fluxes at P_1^{BM} are therefore

$$J_{w,1}^{BM} = n_p\left[0.650\, q_{w,s}^{BM} + 0.350\, q_{w,d}\right] = n_p(2.18 \times 10^{-21}), \tag{18}$$

$$J_{s,1}^{BM} = n_p\left[0.650\, q_{s,s}^{BM}(P_1) + 0.350\, q_{s,d}\right] = n_p(4.02 \times 10^{-19}), \tag{19}$$

which leads to

$$R_1^{BM} = 1.84 \times 10^2. \tag{20}$$

For $P_2 = (0.985{,}0.015)$, the electrostatic factor is

$$K_{\mathrm{elec}}(0.015) = \exp(-2.20 \times 0.015) = \exp(-0.0330) = 0.968. \tag{21}$$

The selective solute term becomes

$$q_{s,s}^{BM}(P_2) = 1.49 \times 10^{-20}. \tag{22}$$

The BM fluxes at P_2 are therefore

$$J_{w,2}^{BM} = n_p\left[0.985\, q_{w,s}^{BM} + 0.015\, q_{w,d}\right] = n_p(4.72 \times 10^{-22}), \tag{23}$$

$$J_{s,2}^{BM} = n_p\left[0.985\, q_{s,s}^{BM}(P_2) + 0.015\, q_{s,d}\right] = n_p(3.16 \times 10^{-20}), \tag{24}$$

which leads to

$$R_2^{BM} = 6.71 \times 10^1. \tag{25}$$

Points P_1 and P_2 are highlighted in Figure S2 to verify that the local colors at those positions are qualitatively consistent with the corresponding values indicated by the color bar.

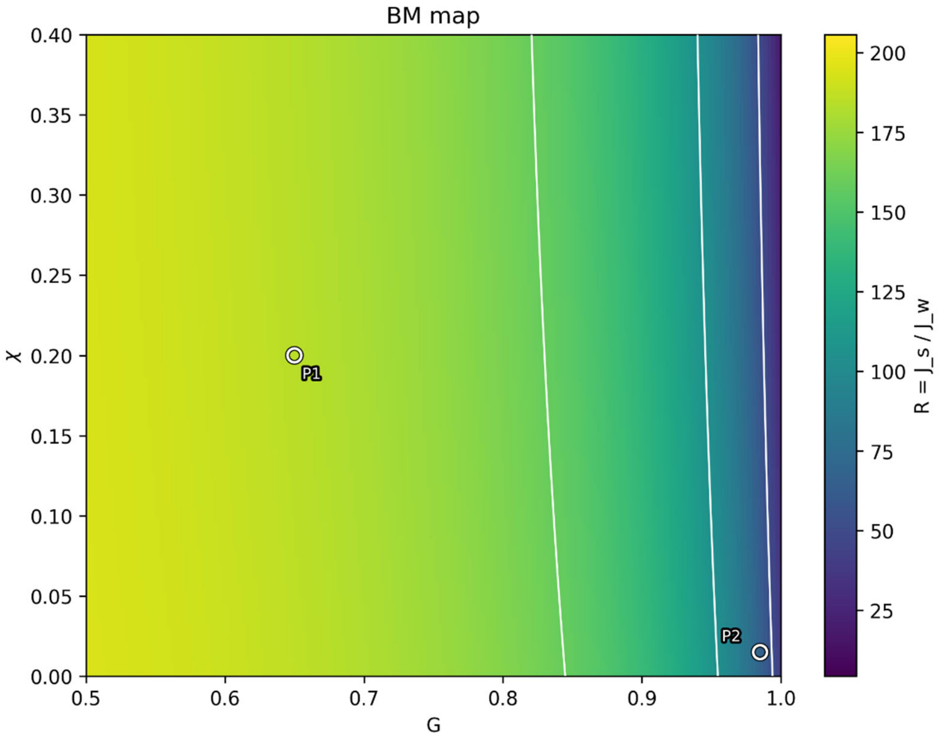

Figure S2. Maps of the coherence ratio $R = J_s/J_w$ in the (G, χ) state space for the BM, EM, and FM formulations. The markers P1 and P2 denote representative checkpoints used as visual consistency tests: the local color at each point is qualitatively consistent with the corresponding value indicated by the color bar. White lines represent selected iso- R contours.

3.3. EM reconstruction at P_1^{EM} and P_2

This section reconstructs the extended model. In EM, chemistry modifies both the selective radius and the effective selective weight through

$$r_{s,\text{eff}} = r_s(1-\beta\chi), G_{\text{eff}} = G(1-\gamma\chi). \quad (26)$$

For $P_1^{EM} = (0.920, 0.050)$, one obtains

$$r_{s,\text{eff},1} = 0.500(1-0.20\times 0.050) = 0.495\,\text{nm}, \quad (27)$$

$$G_{\text{eff},1} = 0.920(1-0.35\times 0.050) = 0.904. \quad (28)$$

The corresponding steric ratio is

$$\lambda_{s,1}^{EM} = \frac{0.325}{0.495} = 0.657, \quad (29)$$

and therefore,

$$K_{\text{steric},s,1}^{EM} = (1-0.657)^2 = 0.118. \quad (30)$$

The electrostatic factor is

$$K_{\text{elec}}(0.050) = \exp(-2.20\times 0.050) = \exp(-0.110) = 0.896. \quad (31)$$

The selective-path hydraulic and solute terms become

$$q_{w,s,1}^{EM} = 3.83\times 10^{-22}, q_{s,s,1}^{EM} = 1.30\times 10^{-20}. \quad (32)$$

The EM fluxes at P_1^{EM} are therefore

$$J_{w,1}^{EM} = n_p\left[0.904\, q_{w,s,1}^{EM} + (1-0.904)\, q_{w,d}\right] = n_p(8.75\times 10^{-22}), \quad (33)$$

$$J_{s,1}^{EM} = n_p\left[0.904\, q_{s,s,1}^{EM} + (1-0.904)\, q_{s,d}\right] = n_p(1.20\times 10^{-19}), \quad (34)$$

which leads to

$$R_1^{EM} = 1.38\times 10^{2}. \quad (35)$$

For $P_2 = (0.985, 0.015)$, one obtains

$$r_{s,\text{eff},2} = 0.500(1-0.20\times 0.015) = 0.498\,\text{nm}, \quad (36)$$

$$G_{\text{eff},2} = 0.985(1-0.35\times 0.015) = 0.980. \quad (37)$$

The corresponding steric ratio is

$$\lambda_{s,2}^{EM} = \frac{0.325}{0.498} = 0.652, \quad (38)$$

and, therefore,

$$K_{\text{steric},s,2}^{EM} = (1-0.652)^2 = 0.121. \quad (39)$$

The electrostatic factor is

$$K_{\text{elec}}(0.015) = 0.968. \quad (40)$$

The selective-path hydraulic and solute terms become

$$q_{w,s,2}^{EM} = 3.92\times 10^{-22}, q_{s,s,2}^{EM} = 1.46\times 10^{-20}. \quad (41)$$

The EM fluxes at P_2 are therefore

$$J_{w,2}^{EM} = n_p\left[0.980\, q_{w,s,2}^{EM} + 0.020\, q_{w,d}\right] = n_p(4.95\times 10^{-22}), \quad (42)$$

$$J_{s,2}^{EM} = n_p\left[0.980\, q_{s,s,2}^{EM} + 0.020\, q_{s,d}\right] = n_p(3.72 \times 10^{-20}), \tag{43}$$

which leads to

$$R_2^{EM} = 7.51 \times 10^1. \tag{44}$$

As expected, the prefactor n_p cancels in the ratio R, so the reconstructed values depend only on the weighted pathway contributions.

Points P_1 and P_2 are highlighted in Figure S3 to verify, as in the BM case, that the local colors at those positions are qualitatively consistent with the corresponding values indicated by the color bar for the EM model.

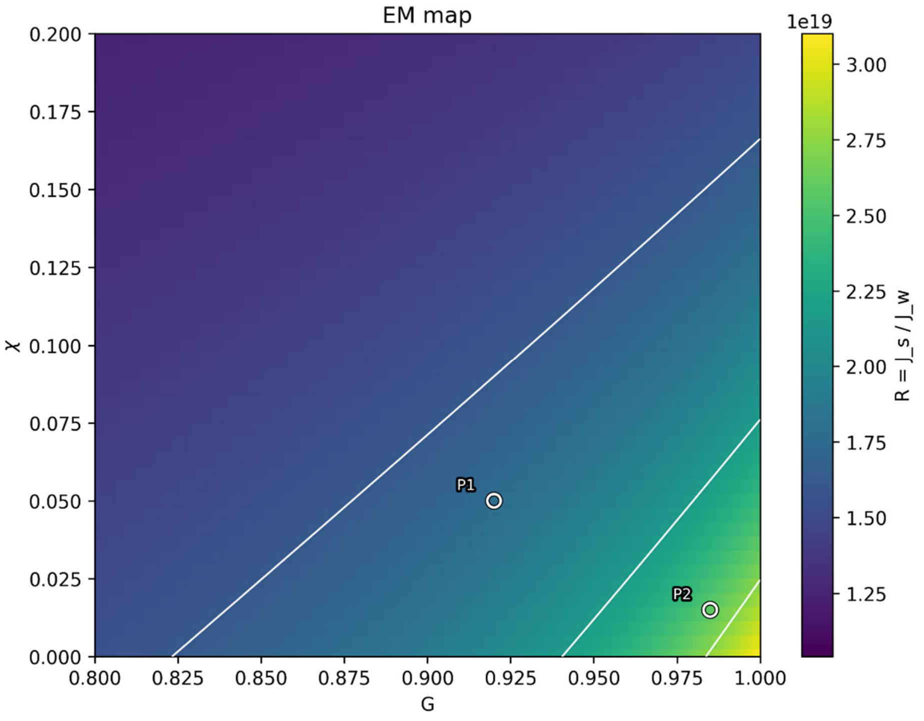

Figure S3. Extended model (EM) map of the coherence ratio $R = J_s/J_w$ as a function of structural selectivity G and nanochemical state χ. Points P1 and P2 indicate representative checkpoints used to verify that the local field color is qualitatively consistent with the corresponding value read from the color bar. White lines denote selected iso- R contours.

3.4. FM reconstruction at P_1^{FM} and P_2

This section reconstructs the full model. In FM, the deterministic EM selective radius is replaced by a normalized selective pore-size distribution, and the selective contribution is evaluated as a distribution-averaged response. For transparency, only the representative weighted sums are reported here; the actual FM reconstruction uses the full processed PSD support.

The support is written as

$$r(u,\chi) = u\, r_{s,\mathrm{eff}}(\chi), \int \rho(u)\, du = 1, \tag{45}$$

with selective averages

$$\bar{q}_{w,s}^{FM}(\chi) = \int q_{w,s}\big(r(u,\chi)\big)\, \rho(u)\, du, \tag{46}$$

$$\bar{q}_{s,s}^{FM}(\chi) = \int q_{s,s}(r(u,\chi),\chi)\, \rho(u)\, du. \tag{47}$$

The full-model fluxes are therefore

$$J_w^{FM} = n_p\left[G_{\text{eff}}\,\bar{q}_{w,s}^{FM} + (1 - G_{\text{eff}})\,q_{w,d}\right], \quad (48)$$

$$J_s^{FM} = n_p\left[G_{\text{eff}}\,\bar{q}_{s,s}^{FM} + (1 - G_{\text{eff}})\,q_{s,d}\right]. \quad (49)$$

For $P_1^{FM} = (0.950, 0.050)$, the weighted selective sums are

$$\bar{q}_{w,s,1}^{FM} = 4.35 \times 10^{-22}, \bar{q}_{s,s,1}^{FM} = 1.79 \times 10^{-20}, G_{\text{eff},1} = 0.933. \quad (50)$$

The FM fluxes are therefore

$$J_{w,1}^{FM} = n_p(7.73 \times 10^{-22}), J_{s,1}^{FM} = n_p(9.20 \times 10^{-20}), R_1^{FM} = 1.19 \times 10^2. \quad (51)$$

For $P_2 = (0.985, 0.015)$, the weighted selective sums are

$$\bar{q}_{w,s,2}^{FM} = 4.44 \times 10^{-22}, \bar{q}_{s,s,2}^{FM} = 2.00 \times 10^{-20}, G_{\text{eff},2} = 0.980. \quad (52)$$

The FM fluxes are therefore

$$J_{w,2}^{FM} = n_p(5.46 \times 10^{-22}), J_{s,2}^{FM} = n_p(4.24 \times 10^{-20}), R_2^{FM} = 7.75 \times 10^1. \quad (53)$$

Points P_1 and P_2 are highlighted in Figure S4 to verify, as in the BM and EM cases, that the local colors at those positions are qualitatively consistent with the corresponding values indicated by the color bar for the FM model.

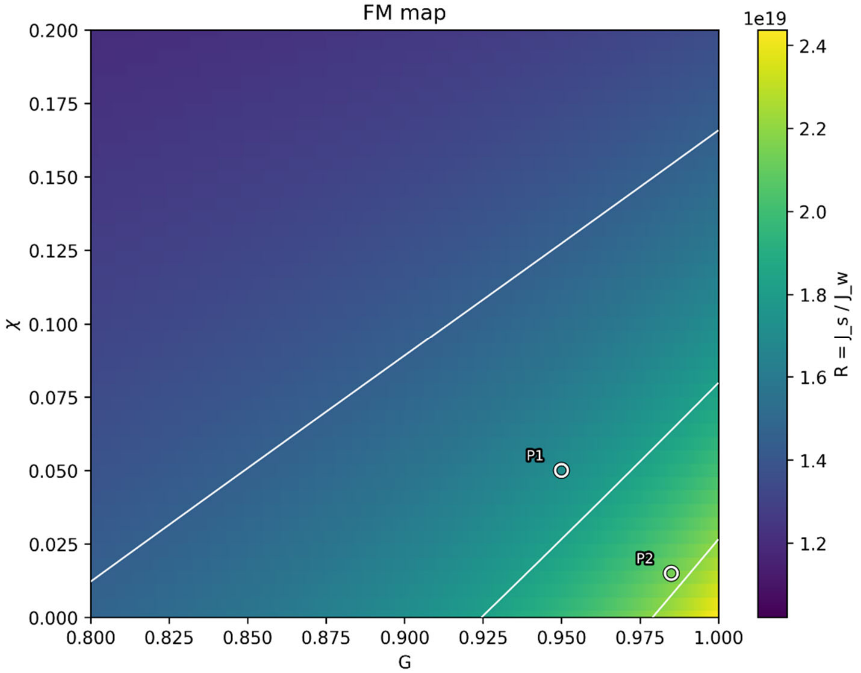

.

Figure S4. FM map of the coherence ratio $R = J_s/J_w$ as a function of G and χ. The points P1 and P2 are included as in Figures S2-S3 as representative checkpoints to verify that the local map color is qualitatively consistent with the associated color-bar value. White lines indicate selected iso- R contours.

3.5. Direct checkpoint comparison and verification against the figures

The reconstructed values may now be checked directly against the corresponding map coordinates and restricted-window panels. For BM, the checkpoint values coincide with direct model evaluation at the stated coordinates. For EM and FM, the graphical verification against the plotted maps uses the nearest sampled array node of the displayed grid.

At the model-specific first checkpoints, the reconstructed values are

$$R_1^{BM} = 1.84 \times 10^2, R_1^{EM} = 1.38 \times 10^2, R_1^{FM} = 1.19 \times 10^2. \quad (54)$$

These model-specific P_1 values are local verification points inside the visually relevant windows of the three panels and are not intended as one-to-one cross-model comparisons.

At the common point $P_2 = (0.985, 0.015)$, the three reconstructed values are

$$R_2^{BM} = 6.71 \times 10^1, R_2^{EM} = 7.51 \times 10^1, R_2^{FM} = 7.75 \times 10^1. \quad (55)$$

The corresponding BM–EM and EM–FM differences are therefore

$$\Delta R_2^{EM-BM} = R_2^{EM} - R_2^{BM} = 8.06, \quad (56)$$

$$\Delta R_2^{FM-EM} = R_2^{FM} - R_2^{EM} = 2.42. \quad (57)$$

A compact summary is thus

$$P_1^{BM}(0.650, 0.200): R^{BM} = 1.84 \times 10^2, \quad (58)$$

$$P_1^{EM}(0.920, 0.050): R^{EM} = 1.38 \times 10^2, \quad (59)$$

$$P_1^{FM}(0.950, 0.050): R^{FM} = 1.19 \times 10^2, \quad (60)$$

$$P_2(0.985, 0.015): R^{BM} = 6.71 \times 10^1, R^{EM} = 7.51 \times 10^1, R^{FM} = 7.75 \times 10^1. \quad (61)$$

These checkpoint choices are fully consistent with the revised plotting windows and with the updated point markers used in the BM, EM, and FM panels.

3.6. Scope of the worked example

This worked example is intended as a transparency and reproducibility exercise. It does not claim a one-to-one inversion from a measured pair to a unique internal coordinate pair. Instead, it shows in explicit numerical form how the BM–EM–FM framework transforms the working parameter set into the transport organization quantified in the figures.

The revised checkpoint structure also clarifies the role of the local tests. The three P_1 values are model-specific verification points placed in the most informative region of each panel, whereas the common point P_2 is the direct BM–EM–FM comparison coordinate. In this sense, the appendix functions as a physically structured consistency check that complements the graphical results and clarifies the computational chain linking the selected parameter set to the reported values.

References

1. Nasr, M.; Alfryyan, N.; Ali, S.S.; Abd El-Salam, H.M.; Shaban, M. Preparation, characterization, and performance of PES/GO woven mixed matrix nanocomposite forward osmosis membrane for water desalination. *RSC Adv.* **2022**, *12*, 25654–25668. https://doi.org/10.1039/D2RA03832C.
2. Sun, P.; Zhu, M.; Wang, K.; Zhong, M.; Wei, J.; Wu, D.; Xu, Z.; Zhu, H. Selective ion penetration of graphene oxide membranes. *ACS Nano* **2013**, *7*, 428–437. https://doi.org/10.1021/nn304471w.
3. Eliseev, A.A.; Gurianov, K.E.; Poyarkov, A.A.; Komkova, M.A.; Sadilov, I.S.; Chumakov, A.P.; Petukhov, D.I. Tunable sieving of ions using graphene oxide: Swelling peculiarities in free-standing and confined states. *Nano Lett.* **2023**, *23*, 9414–9422. https://doi.org/10.1021/acs.nanolett.3c02247.
4. Wan, C.F.; Cui, Y.; Gai, W.X.; Cheng, Z.L.; Chung, T.S. Nanostructured membranes for enhanced forward osmosis and pressure-retarded osmosis. In *Emerging Materials for Sustainable Desalination and Water Remediation*; Elsevier: Amsterdam, The Netherlands, 2020; pp. 373–394. https://doi.org/10.1016/B978-0-12-814681-1.00014-X.

5. Tiwary, S.K.; Singh, M.; Chavan, S.V.; Karim, A. Graphene oxide-based membranes for water desalination and purification. *npj 2D Mater. Appl.* **2024**, *8*, 27. https://doi.org/10.1038/s41699-024-00462-z.
6. Holt, J.K.; Park, H.G.; Wang, Y.; Stadermann, M.; Artyukhin, A.B.; Grigoropoulos, C.P.; Noy, A.; Bakajin, O. Fast mass transport through sub-2-nanometer carbon nanotubes. *Science* **2006**, *312*, 1034–1037. https://doi.org/10.1126/science.1126298.
7. Secchi, E.; Marbach, S.; Niguès, A.; Stein, D.; Siria, A.; Bocquet, L. Massive radius-dependent flow slippage in carbon nanotubes. *Nature* **2016**, *537*, 210–213. https://doi.org/10.1038/nature19315.
8. Wang, P.; Krasavin, A.V.; Nasir, M.E.; Dickson, W.; Zayats, A.V. Reactive tunnel junctions in electrically driven plasmonic nanorod metamaterials. *Nat. Nanotechnol.* **2017**, *13*, 159–164, https://doi.org/10.1038/s41565-017-0017-7.
9. Robinson, R.A; Stokes, R.H. Electrolyte Solutions, 2nd Edition; Butterworths Scientific Publications, London, 1959.
10. Marcus, Y. Ionic radii in aqueous solutions. *Chem. Rev.* **1988**, *88*, 1475–1498, https://doi.org/10.1021/cr00090a003.
11. Schoch, R.B.; Han, J.; Renaud, P. Transport phenomena in nanofluidics. *Rev. Mod. Phys.* **2008**, *80*, 839–883. https://doi.org/10.1103/RevModPhys.80.839.
12. Kavokine, N.; Netz, R.R.; Bocquet, L. Fluids at the nanoscale: From continuum to subcontinuum transport. *Annu. Rev. Fluid Mech.* **2021**, *53*, 377–410. https://doi.org/10.1146/annurev-fluid-071320-095958.
